# An Integrated Database of Small RNAs and Their Interplay With Transcriptional Gene Regulatory Networks in Corynebacteria

**DOI:** 10.3389/fmicb.2021.656435

**Published:** 2021-06-17

**Authors:** Mariana Teixeira Dornelles Parise, Doglas Parise, Flavia Figueira Aburjaile, Anne Cybelle Pinto Gomide, Rodrigo Bentes Kato, Martin Raden, Rolf Backofen, Vasco Ariston de Carvalho Azevedo, Jan Baumbach

**Affiliations:** ^1^Chair of Experimental Bioinformatics, TUM School of Life Sciences, Technical University of Munich, Munich, Germany; ^2^Institute of Biological Sciences, Universidade Federal de Minas Gerais, Belo Horizonte, Brazil; ^3^Bioinformatics, Department of Computer Science, University of Freiburg, Freiburg, Germany; ^4^Computational Biomedicine Lab, Department of Mathematics and Computer Science, University of Southern Denmark, Odense, Denmark; ^5^Chair of Computational Systems Biology, University of Hamburg, Hamburg, Germany

**Keywords:** small RNAs, sRNA targets, *Corynebacterium*, CoryneRegNet, gene regulatory networks

## Abstract

Small RNAs (sRNAs) are one of the key players in the post-transcriptional regulation of bacterial gene expression. These molecules, together with transcription factors, form regulatory networks and greatly influence the bacterial regulatory landscape. Little is known concerning sRNAs and their influence on the regulatory machinery in the genus *Corynebacterium*, despite its medical, veterinary and biotechnological importance. Here, we expand corynebacterial regulatory knowledge by integrating sRNAs and their regulatory interactions into the transcriptional regulatory networks of six corynebacterial species, covering four human and animal pathogens, and integrate this data into the CoryneRegNet database. To this end, we predicted sRNAs to regulate 754 genes, including 206 transcription factors, in corynebacterial gene regulatory networks. Amongst them, the sRNA Cd-NCTC13129-sRNA-2 is predicted to directly regulate *ydfH*, which indirectly regulates 66 genes, including the global regulator *glxR* in *C. diphtheriae*. All of the sRNA-enriched regulatory networks of the genus *Corynebacterium* have been made publicly available in the newest release of CoryneRegNet(www.exbio.wzw.tum.de/coryneregnet/) to aid in providing valuable insights and to guide future experiments.

## Introduction

Small RNAs (sRNAs) have been proven to be important players in the regulatory mechanisms of bacteria (Waters and Storz, 2009; Gripenland et al., 2010; Waters et al., 2017). These molecules interact with messenger RNAs (mRNAs) to induce or repress gene expression post-transcriptionally (De Lay et al., 2013; Papenfort and Vanderpool, 2015). Regulatory sRNAs can both co-regulate genes alongside transcription factors (TFs) and sigma factors, as well as regulate these regulatory proteins, forming regulatory circuits (Lee and Gottesman, 2016; Mandin et al., 2016; Nitzan et al., 2017). Consequently, sRNA regulations have been recently integrated into gene regulatory networks (GRNs), granting these networks a more comprehensive view of gene expression regulation (Beisel and Storz, 2010; Nitzan et al., 2017; Brosse and Guillier, 2018; Arrieta-Ortiz et al., 2020).

Due to its importance, both computational and experimental techniques have been developed for identifying sRNAs and their interactions. Experimental methods such as total RNA labeling (Wu et al., 1996), deep sequencing (Sittka et al., 2008; Sharma and Vogel, 2009; Barquist and Vogel, 2015) and co-immunoprecipitation of RNA-binding proteins (Faner and Feig, 2013) have been used to discover novel sRNAs. Other techniques, such as pulse-expression (Massé et al., 2005), MAPS (Lalaouna and Massé, 2015), RIL-seq (Melamed et al., 2016), and GRIL-seq (Han et al., 2016) have been applied to identify sRNA-mRNA interactions. For a comprehensive description see Altuvia (2007), Ahmed et al. (2018), and Diallo and Provost (2020). Computational methods stand out by revealing promising sRNA candidates for further experimental testing without exhaustive wet-lab assays (Wright and Georg, 2018). In general, sRNA prediction software can be grouped into three types of methods: *de novo*, homology-based and experimental-data dependent (Zhang Y. et al., 2017; Backofen et al., 2018). sRNA target prediction software can be divided into two types of methods: local-interaction based and full-hybrid based (Pain et al., 2015). For further explanations and comparisons of these methods see Pain et al. (2015), Zhang Y. et al. (2017), and Backofen et al. (2018).

Both predicted and experimental bacterial sRNAs have been made publicly available in databases such as Rfam (Kalvari et al., 2018) and RNA central (The RNAcentral Consortium, 2019) for several organisms, including bacteria. Likewise, sRNA data for Gram-positive bacteria is available on sRNAdb (Pischimarov et al., 2012). BSRD (Li et al., 2013), sRNATarBase (Wang et al., 2016), sRNAMap (Huang et al., 2009), and RNAInter (Lin et al., 2020) provide sRNA regulatory information for several bacterial species. Despite the influence and importance of these molecules on gene expression, databases integrating sRNA-based and transcriptional regulatory networks are largely missing. To the best of our knowledge, RegulonDB (Santos-Zavaleta et al., 2019), the reference database for *Escherichia coli* GRNs, is the only one to have done this integration though exclusively for *E. coli* K12.

In the context of the *Corynebacterium* genus, CoryneRegNet (Parise et al., 2020) is the reference database for Corynebacterial transcriptional regulatory networks, containing more than 80,000 regulatory interactions but lacking sRNA data. A few Corynebacterial sRNAs can be found in BSRD (Li et al., 2013), Rfam (Kalvari et al., 2018), and RNA central (The RNAcentral Consortium, 2019). For *Corynebacterium glutamicium*, the model organism for this genus, 805 sRNAs were experimentally identified using deep sequencing and were reported in Mentz et al. (2013). However, there are no experimental or predicted sRNA regulations for the *Corynebacterium* genus.

Here, we present the first study about the integration of sRNA regulations with transcriptional regulation in corynebacteria. We predicted sRNAs and their targets for six *Corynebacterium* species of either medical, veterinary or industrial interest, yielding 922 sRNAs and 6,389 sRNA regulatory interactions. This data was integrated into CoryneRegNet 7.5, revealing 754 genes in the GRN to be regulated by both sRNAs and transcription factors and 206 regulatory proteins to be regulated by sRNAs. In a case study of human pathogenic corynebacteria using the CoryneRegNet 7.5 sRNA-enriched database content, we predict the sRNAS *Cd-NCTC13129-sRNA-2* and *scjk1464.1* to form regulatory cascades with TFs. *Cd-NCTC13129-sRNA-2* is predicted to regulate the *ydfH* homolog, indirectly regulating 66 genes in *C. diphtheriae* and *scjk1464.1* is predicted to regulate *mcbR* and *dtxR*, indirectly regulating 35 genes in *C. jeikeium*. In the animal pathogen *C. pseudotuberculosis*, the virulence factor *fagC* is also predicted to be regulated by the sRNA *Cp-1002B-sRNA-1*. To sum up, the integration of sRNAs and their interactions into the transcriptional regulatory networks in CoryneRegNet provides a more comprehensive view on corynebacterial regulatory mechanisms.

## Materials and Methods

**FIGURE 1:**
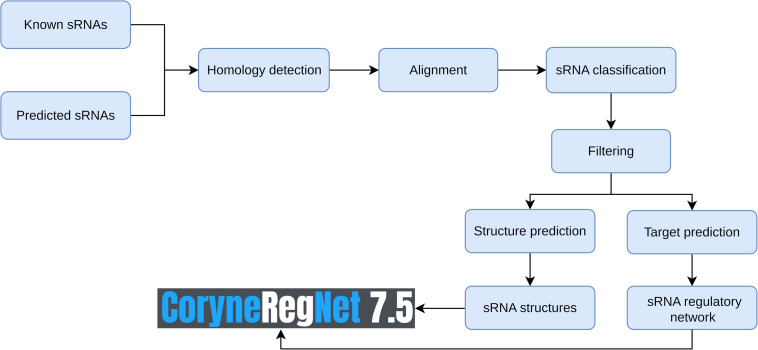
Overview of the sRNA data integration workflow.

The CoryneRegNet sRNA integration pipeline consists of seven steps: sRNA collection and prediction, homology detection, alignment, sRNA classification, filter, structure prediction and target prediction. An overview of these steps is shown in Figure 1. We started with compiling a dataset of 805 experimentally verified sRNAs from Mentz et al. (2013) and 70 predicted sRNAs from BSRD (Li et al., 2013). In order to predict novel sRNAs, we used cmsearch (Nawrocki and Eddy, 2013) on the target genomes with no experimental sRNAs publicly available. Details about the sRNA datasets and the genomes used in this analysis are given in Table 1.

**TABLE 1:** The sRNA datasets and target species.

Strain	Accession number	sRNA dataset		
		Mentz et al., 2013	BSRD	This study
*Corynebacterium diphtheriae* NCTC 13129	NC_002935.2		x	x
*Corynebacterium efficiens* YS-314	NC_004369.1		x	x
*Corynebacterium glutamicum* ATCC 13032	BX927147.1	x	x	
*Corynebacterium jeikeium* K411	NC_007164.1		x	x
*Corynebacterium ulcerans* NCTC7910	NZ_LS483400.1			x
*Corynebacterium pseudotuberculosis* 1002B	NZ_CP012837.1			x

Afterward, we identified homologs for every sRNA in the analysis by using GLASSgo (Lott et al., 2018). Homologous sRNAs belonging to the genomes of interest were incorporated into the analysis. For each sRNA in the analysis, we selected its most distant homologs from the same species and from the same genus with ≥80% of similarity. Thus, these sequences were aligned by using clustalo (Sievers et al., 2011). The sRNAs were classified as either functional or non-functional by running RNAz (Gruber et al., 2010) and RNAdetect (Chen et al., 2019) based on the stability and the conservation of the predicted RNA structures as well as on sequence homology. Predicted sRNAs that were classified as non-functional were removed from the analysis. The secondary structure was predicted using RNAalifold (Bernhart et al., 2008) for every sRNA in the analysis. Furthermore, sRNA targets were predicted by running CopraRNA (Wright et al., 2013) with default settings. Adjusted *p*-values were calculated using the Beijamini-Hochberg correction from the R package stats, method p.adjust (Stats, 2020). Then, we selected the fifteen best-ranked interactions predicted with a *p*-value < 0.01, as suggested in Wright and Georg (2018). The sRNAs and their targets were integrated into CoryneRegNet (Parise et al., 2020) by updating the front-end and back-end, as well as the database. Finally, we predicted gene ontologies for every gene regulated by sRNAs by running Go Feat (Araujo et al., 2018). A detailed explanation of these methods as well as an example can be seen in the Supplementary Material, section II.

## Results

### Database Content

We presented CoryneRegNet 7.5, an updated release of the corynebacterial reference database and analysis platform, now including sRNA networks integrated with the transcriptional regulatory networks of the genus *Corynebacterium*. A total of 922 sRNAs and 6,389 regulatory interactions for six corynebacterial strains were integrated into our database, as shown in Table 2. In total, CoryneRegNet release 7.5 now holds 88,657 regulatory interactions, 10,077 regulators and 59,848 regulated genes. The updated database content is publicly available on CoryneRegNet’s download page:

**TABLE 2:** New, sRNA-related database content of CoryneRegNet 7.5.

Strain	sRNA		sRNA regulatory interaction
		
	Experimental	Predicted	Predicted
*Corynebacterium diphtheriae* NCTC 13129	−	19	176
*Corynebacterium efficiens* YS-314	−	44	439
*Corynebacterium glutamicum* ATCC 13032	805	17	5,324
*Corynebacterium jeikeium* K411	−	27	343
*Corynebacterium ulcerans* NCTC7910	−	6	65
*Corynebacterium pseudotuberculosis* 1002B	−	4	42
Total	805	117	6,399

https://www.exbio.wzw.tum.de/coryneregnet/processToDownload.htm.

### Website

**FIGURE 2:**
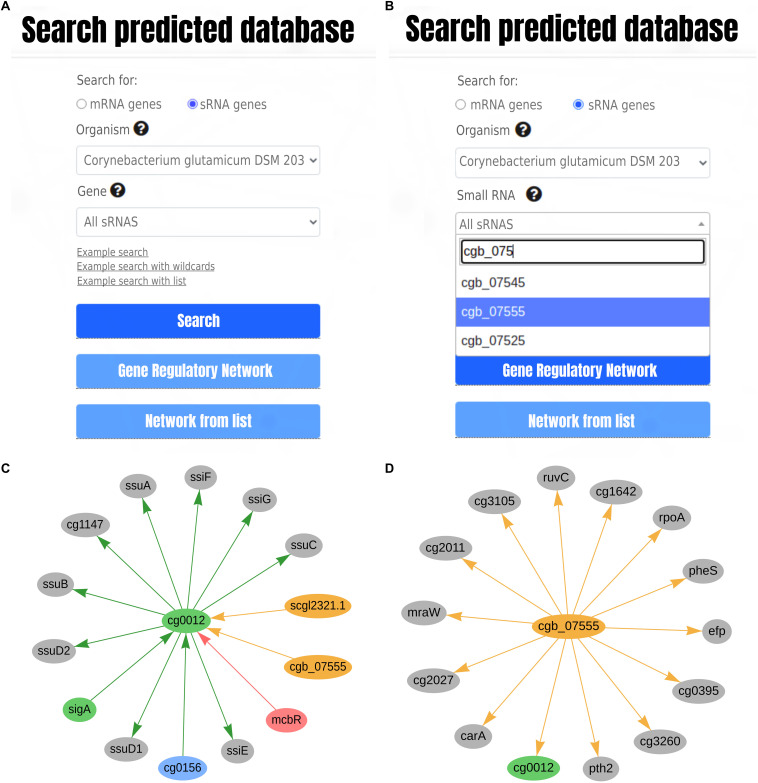
CoryneRegNet’s front-end updates in **(A,B)** search page and **(C,D)** in the network visualization. **(A)** The search page of CoryneRegNet’s database allows for choosing between searching for mRNA genes or sRNA genes while **(B)** guiding the search with gene or sRNA identifiers. **(C)** Direct regulations of cg0012 and **(D)** genes regulated by cgb_07555. In the network, green nodes represent activator proteins, red nodes represent repressor proteins, blue nodes represent dual regulators (i.e., that can activate and repress gene expression), orange nodes represent sRNAs and gray nodes represent target genes. The arrows represent the regulatory interactions and their colors represent the same roles as in the nodes.

We updated CoryneRegNet’s user interface to present information concerning sRNAs and their targets. Both the regulatory interaction table view and the network view were updated and enriched with corresponding sRNA-related features. The search page now allows the user to (i) search for gene identifiers (Figure 2B) when querying the database for mRNA or sRNA genes (Figure 2A) and (ii) search for a list of genes.

Depending on the search choice (Figure 2A), the user will be directed to the gene-centered or sRNA-centered network view, as presented in Figures 2C,D, respectively. sRNAs and their regulatory interactions have been integrated into the network visualization as orange nodes and directed edges. Considering there is no annotation of activation/repression prediction for the sRNA-mRNA interactions, we represent every sRNA regulatory interaction as an orange, directed edge. The complete sRNA-mRNA interactions set of a genome can also be visualized in case no specific gene or sRNA is selected.

In addition, users can now find genes and sRNAs of interest by using the new filtering and sorting features in the table-oriented view, as presented in Supplementary Figures 1A,B, respectively. In the sRNA view, we included filters for: (i) sRNAs regulating transcription factors, (ii) sRNAs regulating genes in the TRN, and (iii) functional sRNAs. Likewise, in the gene view we included filters for: (i) genes encoding regulatory proteins, (ii) genes regulated by regulatory proteins, (iii) genes regulated by sRNAs, and (iv) genes regulated by sRNAs and/or regulatory proteins.

**FIGURE 3:**
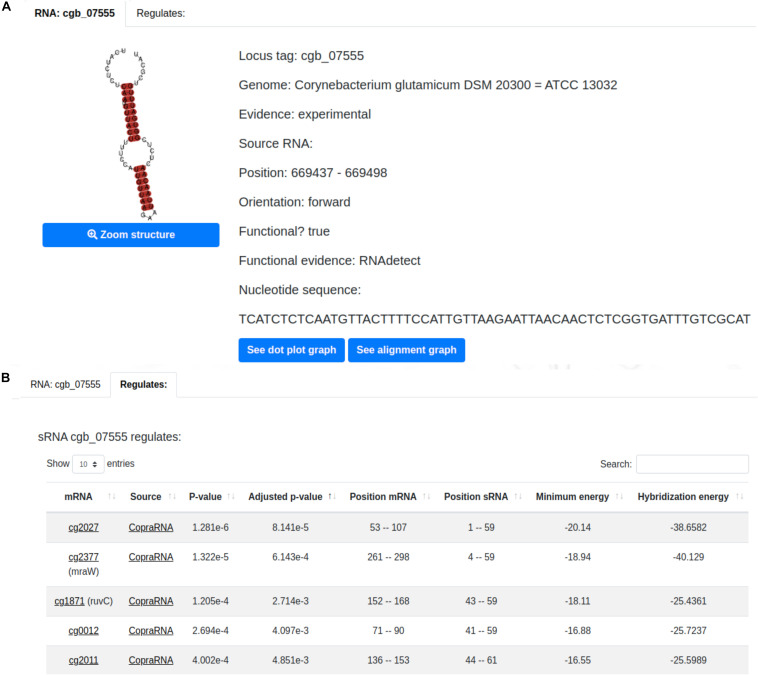
CoryneRegNet 7.5’s sRNA details page with **(A)** essential information of the sRNA cgb_07555 and **(B)** its regulations.

A sample sRNA page is displayed in Figure 3A. It presents essential information of the sRNA of interest such as: type of evidence, position and orientation in the genome, whether or not the sRNA was classified as functional, and the sRNAs’ nucleotide sequence. The predicted structure of the selected sRNA is also presented along with its dot plot and alignment graph. The former illustrates the interaction between the nucleotides (Supplementary Figure 2A) and the latter the conservation between the sRNA of interest and its homologous sRNAs (Supplementary Figure 2B). Additionally, the user can visualize the sRNA regulatory interactions in the “Regulates” tab (Figure 3B). This tab shows information regarding each regulatory interaction predicted by CopraRNA (Wright et al., 2013) of the selected sRNA such as its position, minimum energy, hybridization energy and *p*-value.

Furthermore, we integrated the sRNA interaction network into the statistics section with three new analyses: (i) quantities of sRNA types (Supplementary Figure 3A), (ii) distribution of sRNAs regulating a gene (Supplementary Figure 3C), and (iii) distribution of co-regulating sRNAs (Supplementary Figure 3B). Finally, we updated the documentation and workflow sections at the website accordingly.

### Case Study

We illustrate the utility of the sRNA-enriched CoryneRegNet 7.5 by utilizing the updated filtering features to identify 206 regulatory proteins regulated by sRNAs and 754 genes regulated by both sRNAs and TFs in our six genomes. We selected the genes regulated by both sRNAs and TFs in the following four pathogenic bacteria: *C. diphtheriae* NCTC 13129, *C. jeikeium* K411, *C. pseudotuberculosis* 1002B and *C. ulcerans* NCTC7910. In addition, we selected gene circuits in these pathogenic bacteria and in the model organism *C. glutamicum* and presented whether these observations are conserved in *C. efficiens*. We visualized the regulatory networks of these genes using the list-based network feature in CoryneRegNet 7.5, where we also collected their homologous genes.

**FIGURE 4:**
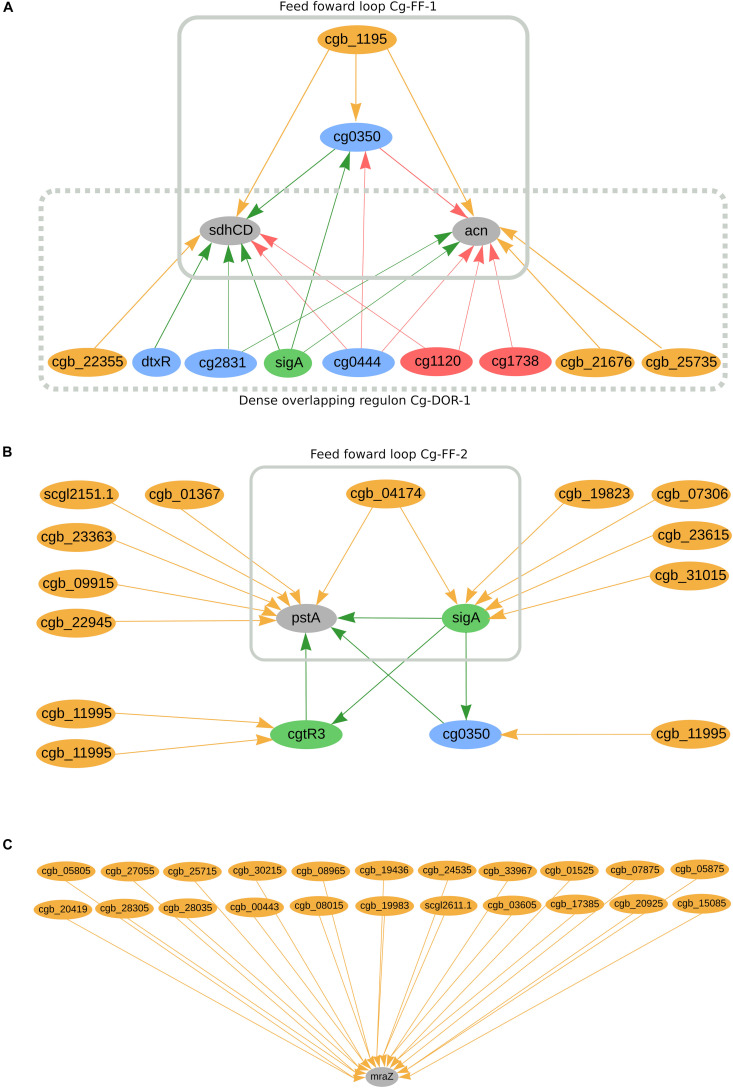
*C. glutamicum*’s predicted sRNA-enriched regulons. **(A)** *sdhCC* and *acn* co-regulated by TFs and sRNAs and forming two regulatory circuits, Cg-DOR-1 and Cg-FF-1. **(B)** pstA being directly and indirectly regulated by TFs and sRNAs, forming the regulatory circuit Cg-FF-2. **(C)** marZ being regulated by 22 sRNAs. In the networks, green nodes represent activator proteins, red nodes represent repressor proteins, blue nodes represent dual regulators (i.e., that can activate and repress gene expression), orange nodes represent sRNAs and gray nodes represent target genes. The arrows represent the regulatory interactions and their colors represent the same roles as the ones in the nodes.

In *C. glutamicum*, we predicted 662 genes to be co-regulated by sRNAs and TFs. Amongst them, we can highlight cg0350, *sdhCD*, *acn*, *cgtR3*, *pstA*, and the sigma factor *sigA*, as presented in Figure 4A. The sRNA cgb_1195 potentially co-regulates cg0350 (*glxR* homolog) together with four transcriptional regulators: cg2544 (*ydfH* homolog), cg0146 (*sucR* homolog), *sigA*, and cg0444 (*ramB* homolog). Additionally, cg0350 has been reported to regulate itself in this organism. The sRNA is predicted to directly and indirectly regulate the highly regulated genes *sdhCD* and *acn*, forming feed forward loop Cg-FF-1 (Figure 4A). These two genes are also part of the dense overlapping regulon Cg-DOR-1, in which three other sRNAs potentially co-regulate them together with five TFs and *sigA*. The membrane anchor subunit *sdhCD* jointly encodes with *sdhA* and *sdhB* the succinate dehydrogenase enzyme, a component of the TCA cycle (Polen et al., 2007; Bussmann et al., 2009). The *acn* gene is also a component of the TCA cycle; it encodes an aconitase enzyme and its inactivation is detrimental to cell growth (Yoon and Woo, 2018). Both the *sdhCD* and *acn* genes were found differentially expressed in acetate medium when compared with glucose medium (Bott, 2007). Figure 4B presents the highly regulated *pstA* as being potentially co-regulated by six sRNAs, two transcription factors and *sigA*. The sRNA cgb_04174 is predicted to directly and indirectly regulate *pstA*, forming the feed forward loop Cg-FF-2. In total, *pstA* is predicted to be directly regulated by six sRNAs and indirectly regulated by eigth sRNAs. This gene is part of the Pst system, which is part of the inorganic orthophosphate (P_*i*_) starvation stimulon in *C. glutamicum* (Ishige et al., 2003). The transcriptional regulators *sigA*, *cgtR3* and cg0350 are also predicted to be regulated by sRNAs. *SigA* is the primary sigma factor in *C. glutamicum* and is potentially regulated by five sRNAs; this regulator is considered responsible for the transcription of the majority of the housekeeping genes in this organism (Oguiza et al., 1996; Schröder and Tauch, 2010). The global regulator cg0350 (*glxR* homolog) has been reported to be involved in the regulation of 195 genes in *C. glutamicum* (Freyre-González and Tauch, 2017; Parise et al., 2020) and is potentially regulated by one sRNA. The regulator *cgtR3* (*phoR*) is the master regulator of phosphate metabolism in *C. glutamicum* and is potentially regulated by two sRNAs (Schröder and Tauch, 2010). None of the observations mentioned so far is conserved in the other organisms analyzed in this study. Furthermore, *mraZ* is predicted to be regulated by 22 sRNAs, as presented in Figure 4C. This gene is highly conserved in bacteria and is part of the division cell cluster (*dcw*) (Eraso et al., 2014). The cleavage of the coding region of its mRNA is required for efficient cell division in *C. glutamicum* (Maeda et al., 2016). The other genes from the *mraZ* operon, *mraW*, and cg2376 (*ftsL* homolog), are potentially regulated by sRNAs. *MraW* is potentially regulated by six sRNAs; amongst them, cgb_03605 is also predicted to regulate *mraZ*. Cg2376 is predicted to be regulated by one sRNA. *MraZ* homolog genes in *C. efficiens*, *C. jeikeium*, and *C. pseudotuberculosis* are also potentially regulated by 10 sRNAs, two sRNAs and one sRNA, respectively. In *C. ulcerans*, the *mraW* homolog is potentially regulated by one sRNA, whereas none of the cg2376 homologs are predicted to be regulated by sRNAs in this study.

**FIGURE 5:**
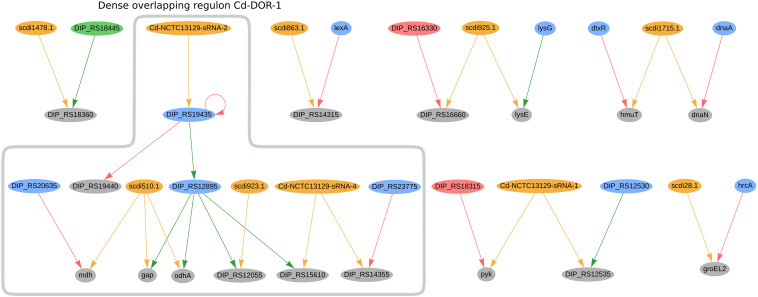
Genes regulated by sRNAs and regulatory proteins in *C. diphtheriae* NCTC 13129. In the network, green nodes represent activator proteins, red nodes represent repressor proteins, blue nodes represent dual regulators (i.e., that can activate and repress gene expression), orange nodes represent sRNAs and gray nodes represent target genes. The arrows represent the regulatory interactions and their colors represent the same roles as the ones in the nodes.

In *C. diphtheriae* NCTC 13129, we predicted 16 genes to be co-regulated by sRNAs and TFs; the regulatory network of these genes can be seen in Figure 5. Amongst them, the sRNA Cd-NCTC13129-sRNA-2 potentially regulates the transcription factor DIP_RS19435 (*ydfH* homolog), forming a single-input module inside the dense overlapping regulon Cd-DOR-1 (Figure 5). The *ydfH* homolog is predicted to auto-regulate itself and to regulate DIP_RS12895 (*glxR* homolog). It forms a regulatory cascade where the complete set of genes regulated by *glx*R may be indirectly regulated by this sRNA, accounting for 66 genes. The complete regulon of *ydfH* and *glxR* is presented in Supplementary Figure 4. As presented in the dense overlapping regulon Cd-DOR-1 (Figure 5), the GlxR homolog TF potentially co-regulates four genes with sRNAs: DIP_RS15610 (*ispE* homolog), *gap*, *odhA* and DIP_RS12055. The sRNA Cd-NCTC13129-sRNA-4 potentially regulates both the *ispE* homolog and DIP_RS14355, a methionine ABC transporter substrate-binding. The latter is also regulated by the TetR/AcrR-family regulator DIP_RS23775 (*mcbR* homolog). In C. efficiens, the homologous methionine ABC transporter substrate-binding (CE_RS03295) is also potentially co-regulated by one sRNA (Ce-YS314-sRNA-28) and a TetR/AcrR family TF (CE_RS13790). Also in Cd-DOR-1 (Figure 5), *gap* and *odhA* are predicted to be regulated by the same sRNA, scdi510.1, which also co-regulates *mdh* along with the LuxR family regulator DIP_RS20635 (*ramA* homolog). Likewise, *gap* (cg1791) is also predicted to be co-regulated by cg0350 (*glxR* homolog) and the sRNAs scgl2151.1, cgb_23426 and cgb_10355 in *C. glutamicum*. In general, the genes in Cd-DOR-1 are involved in the TCA cycle and in carbohydrate metabolism.

Also in *C. diphtheriae*, five other genes are potentially co-regulated by both sRNAs and TFs. The hemin-binding protein *hmuT* (Draganova et al., 2015) is potentially co-regulated by scdi175.1 and *dtxR*. The sRNA scdi28.1 is predicted to co-regulate the heat-shock protein GroEL2 along with the transcription factor *hrcA*. In *C. efficiens*, the GroEL2 homolog (CE_RS12690) is also predicted to be regulated by a sRNA (Ce-YS314-sRNA-3) and a *hrcA* homolog (CE_RS10870). In *C. diphtheriae*, Cd-NCTC13129-sRNA1 potentially regulates DIP_RS12535 (*pdxS* homolog) and *pyk*, which are also regulated by DIP_RS18315 (*gatR* homolog) and DIP_RS12530 (*pdxR* homolog), respectively. We also observed the DIP_RS18360 gene (*hflX* homolog) being potentially co-regulated by an XRE family transcriptional regulator and the sRNA scdi1478.1.

**FIGURE 6:**
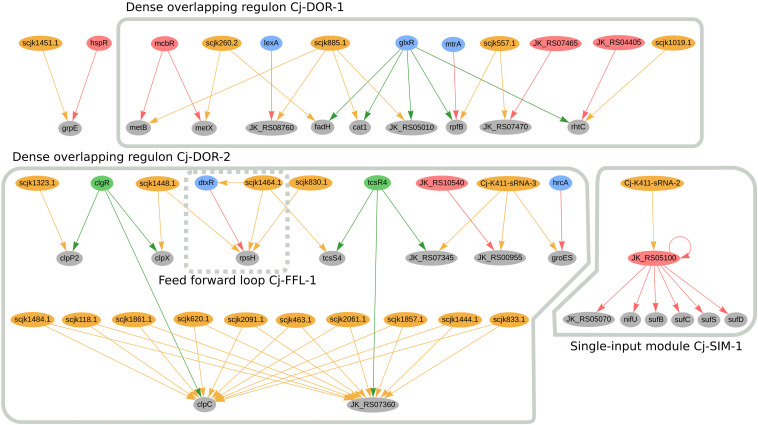
Genes regulated by sRNAs and regulatory proteins in *C. jeikeium* K411. In the network, green nodes represent activator proteins, red nodes represent repressor proteins, blue nodes represent dual regulators (i.e., that can activate and repress gene expression), orange nodes represent sRNAs and gray nodes represent target genes. The arrows represent the regulatory interactions and their colors represent the same roles as the ones in the nodes.

In *C. jeikeium* K411, we predicted twenty genes to be jointly regulated by sRNAs and TFs; the regulatory network of these genes is presented in Figure 6. Amongst these genes we identified two dense overlapping regulons, highlighted as Cj-DOR-1 and Cj-DOR-2. In Cj-DOR-1, the sRNAs scjk260.2, scjk885.1, scjk557.1, scjk1019.1 are predicted to co-regulate five genes (*rhtC*, *fadH*, *rpfB*, *cat1*, and JK_RS05010) with the global regulator *glxR*. The gene JK_RS05010 (*rpfI* homolog) was predicted to have hydrolase activity and is potentially co-regulated by *glxR*, *mtrA* and scjk577.1. The *rpfI* gene, which encodes a resuscitation-promoting factor interacting protein, is a virulence factor in *C. ulcerans* (Trost et al., 2011). The deletion of this gene impaired the growth of long-stored cells in *C. glutamicum* (Hartmann et al., 2004). The other resuscitation-promoting factor, *rpfB*, is also potentially regulated by *mtrA*. In *C. efficiens*, the *rpfB* homolog is also potentially co-regulated by the sRNA Ce-YS314-sRNA-12, the *glxR* homolog (CE_RS01675) and the *mtrA* homolog (CE_RS03955). Also in Cj-DOR-1, *metB* and *metX* are potentially co-regulated by *metR* and one sRNA, these genes are involved in the metabolism of methionine in *C. glutamicum* (Rückert et al., 2003). In the single-input module Cj-SIM-1, the sRNA Cj-K411-sRNA2 potentially regulates the transcription factor JK_RS05100 (*sufR* homolog), indirectly regulating the *sufBDCS* gene cluster and the *nif* operon (*nifU*-JK_RS05070). The genes in this circuit are involved in the formation of iron-sulfur clusters in bacteria (Frazzon, 2003; Outten and Wayne Outten, 2015). In *C. efficiens*, the *sufR* homolog (CE_RS08375) is also potentially regulated by two sRNAs (scef1290.1 and scef1536.1) and regulates the *nif* operon (*nifU*-CE_RS08405) as well as the *sufBDCS* gene cluster (CE_RS08400, CE_RS08395, CE_RS08390, CE_RS08385).

Cj-DOR-2 (Figure 6) contains a cluster of 10 sRNAs potentially co-regulating two genes along with the transcription factors TcsR4 and ClgR. When analyzing these sRNAs, we noticed sRNAs scjk2061.1, scjk118.1, scjk463.1, scjk1484.1, scjk1444.1, scjk2091.1, scjk1857.1, scjk1861.1, scjk620.1, and scjk833.1 are identical copies of the same sRNA located in different regions of the genome. The genomic coordinates of these sRNAs are presented in Supplementary Table III. the following regions of the genome: 117083–117197, 462452–462566, 619808–619922, 832580–832694, 1443235–1443349, 1483232–1483346, 1856182–1856296, 1860886–1861000, 2060398–2060.512, 2090313–2090427. The genes potentially regulated by these sRNAs, *clpC*, and JK_RS07360, encode a Clp ATPase subunit and a hypothetical protein, respectively. In addition to regulating *clpC*, ClgR is also predicted to co-regulate two other genes with sRNAs, *clpP2* and *clpX.* Both *clpC* and *clpP2* are part of a protein quality control system of the cell along with the other proteolytic subunit *clpP1* (Schröder and Tauch, 2010). *ClpX* is also an ATPase subunit that belongs to the Clp/Hsp100 superfamily, which is involved in stress response, energy metabolism, NADPH synthesis and glucose consumption (Huang et al., 2020). This observation is not conserved amongst the Corynebacterial species analyzed in this manuscript. In Cj-DOR-2, the sRNA scjk1464.1 and *tscR4* potentially co-regulate the sensor histidine kinase *tcsS4*, which belongs to a two-component signal transduction system. These systems are important to bacteria due to their capacity to detect and adapt to changes in the environment (Pao and Saier, 1995). *TscR4* is also predicted to regulate the copper chaperone JK_RS07345 alongside the sRNA Cj-K411-sRNA-3. Likewise, this sRNA potentially co-regulates the heat shock protein *groES* and the flavin-dependent oxidoreductase JK_RS00955, which are also regulated by the *hrcA* and JK_RS10540 (*maR1* homolog), respectively. GroES is involved in the transport of proteins and in the post-translational folding, along with the heat shock protein GroEL (Rinke et al., 1992). In general, genes in Cj-DOR-2 are potentially involved in growth and cell proliferation.

In *C. jeikeium*, the diphtheria toxin repressor DtxR, regulates many genes associated with iron metabolism and forms the feed forward loop Cj-FFL-1 with the sRNA scjk1464.1 by directly and indirectly regulating *rpsH* (Figure 6). This sRNA is also predicted to directly regulate the transcription factor *mcbR* (Supplementary Figure 5). By potentially regulating *mcbR* and *dtxR*, scjk1464.1 is predicted to indirectly regulate thirty-five genes. Additionally, two other sRNAs (scjk830.1 and scjk1448.1) are predicted to regulate *rpsH*. This gene encodes a 30S ribosomal protein that is associated with the small ribosomal subunit and has been considered as a potential drug target in *C. diphtheriae* (Jamal et al., 2017; Hassan et al., 2018). By analyzing these sRNAs in Rfam, we observed that they do not belong to the same sRNA family. Furthermore, the sRNA scjk1019 is predicted to co-regulate *rhtC* with *glxR* and JK_04405 (*argR* homolog). This gene was used to increase the production of L-threonine in *C. glutamicum* (Diesveld et al., 2009).

**FIGURE 7:**
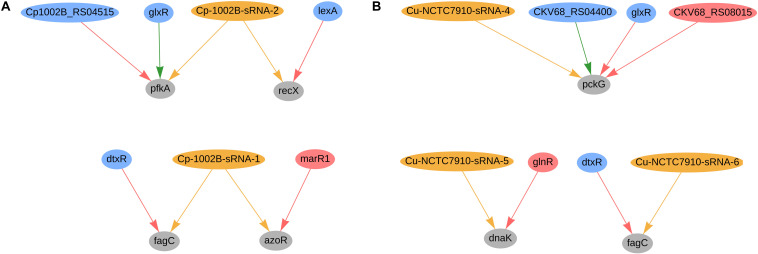
Genes regulated by sRNAs and regulatory proteins in *C. pseudotuberculosis* 1002B **(A)** and in *C. ulcerans* **(B)**. In the network, green nodes represent activator proteins, red nodes represent repressor proteins, blue nodes represent dual regulators (i.e., that can activate and repress gene expression), orange nodes represent sRNAs and gray nodes represent target genes. The arrows represent the regulatory interactions and its colors represent the same roles as the ones in the nodes.

In *C. pseudotuberculosis* 1002B, four genes were predicted to be co-regulated by sRNAs and TFs; the regulatory network of these genes is presented in Figure 7A. The *fagC* (Cp1002B_RS00130) gene is potentially regulated by sRNA Cp-1002B-sRNA-1, as well by the diphtheria toxin repressor (*dtxR*), and is part of the operon *fagABC*. This operon is an active part of the iron acquisition system and is a known virulence factor in *C. pseudotuberculosis* (Billington et al., 2002). Likewise, *fagC* is also potentially regulated by one sRNA (Cu-NCTC7910-sRNA-6) and *dtxR* (CKV68_RS01925) in *C. ulcerans*, as shown in Figure 7B. In *C. pseudotuberculosis*, Cp-1002B-sRNA-1 potentially co-regulates the *azoR* gene along with *marR1*; this gene encodes a flavin mononucleotide (FMN)-dependent homodimeric azobenzene reductase and is involved in the response of oxidative stress. In *C. efficiens*, the a*zoR* homolog (CE_RS08755) is also potentially regulated by one sRNA (scef1673.1) and the *marR1* homolog (CE_RS06390), whereas in *C. glutamicum*, the a*zoR* homolog (cg1850) is potentially regulated by three sRNAs (cgb_31975, cgb_30915, and scgl2371.1) and the *marR1* homolog (cg1324). In *C. pseudotuberculosis* (Figure 7A), the gene *pfkA* (phosphofructokinase) is predicted to be regulated by Cp-1002B-sRNA-2, *glxR*, and Cp1002B_RS04515 (*ramA* homolog). This gene is involved in the reduction of the amount of fructose-6-phosphate during the L-serine fermentation process with sucrose as a carbon resource in *C. glutamicum* (Zhang X. et al., 2017). The *PfkA* homolog in *C. glutamicum* is also potentially regulated by sRNAs and TFs, as presented in Figure 4A. Also in *C. pseudotuberculosis*, Cp-1002B-sRNA-2 also regulates the *recX* gene along with LexA; both *lexA* and *recX* are involved in the bacterial SOS response, acting in DNA damage repair (Pogson et al., 1996; Jochmann et al., 2009; Resende et al., 2011). In *C. glutamicum*, the *recX* homolog (cg2140) is also potentially regulated by two sRNAs (cgb_10545 and cgb_17865) and the *lexA* homolog (cg2114).

In *C. ulcerans* NCTC7910, we also predicted other 2 genes to be regulated by sRNAs and TFs; the regulatory network of these genes is presented in Figure 7B. The *pckG* gene, which encodes a phosphoenolpyruvate carboxykinase, was predicted to be regulated by one sRNA and three transcription factors (*glxR*, *ramA*, and *ramB*). The transcription factor *DnaK* is regulated by one sRNA and the transcription factor *glnR*. Additionally, it regulates the expression of both genes involved in bacterial adhesion and virulence factors in other bacteria (Hanawa et al., 2002; Gomide et al., 2018). These observations are not conserved in the other genomes analyzed in this study.

## Discussion

Although several databases on sRNAs and GRNs exist, the integration of these regulatory networks is still a missing point in deciphering gene expression. Several studies have shown the interplay between TFs and sRNAs when regulating gene expression by forming regulatory circuits, as reviewed by Beisel and Storz (2010); Nitzan et al. (2017), Brosse and Guillier (2018). Furthermore, consistency assessments in *E. coli* (Larsen et al., 2019) and *C. glutamicum* (Parise et al., 2021) showed that regulation driven by transcription factors is not able to satisfactorily explain gene expression and suggested other layers of regulation to be integrated into the networks in order to model the complexity of gene expression. Our work contributes to expanding the regulatory landscape of two biotechnological and four pathogenic *Corynebacterium* species by predicting their sRNA regulatory networks and by integrating them into the corresponding GRNs.

Regarding sRNA prediction, we searched for (i) sRNA homologous of the experimentally validated ones from Mentz et al. (2013) using GLASSgo (Lott et al., 2018) and (ii) novel sRNAs belonging to known sRNA families from Rfam (Kalvari et al., 2021) using cmsearch (Nawrocki and Eddy, 2013). The former uses iterative blast search, pairwise identity filtering and graph-based clustering based on secondary structures to find sRNA homologous (Lott et al., 2018). It allows us to search for homologous sRNAs not belonging to a specific sRNA family. Meanwhile, cmsearch allows us to use covariance models to search for novel members of curated sRNA families from Rfam. Cmsearch has been considered the most specific and sensitive sRNA homology tool (Freyhult et al., 2007; Lott et al., 2018) and GLASSgo presented results comparable to cmsearch in a recent benchmark (Lott et al., 2018). RNAz and RNAdetect identify functional sRNA candidates amongst the ones predicted by GLASSgo and cmsearch, yielding strong candidates for further investigation as well as target prediction (Gruber et al., 2010; Backofen et al., 2018; Chen et al., 2019). Regarding the sRNA target prediction, CopraRNA is currently considered the best bacterial sRNA-mRNA interaction prediction software (Pain et al., 2015; Georg et al., 2020). It constructs a combined prediction based on the conservation of sRNA interactions across a given set of organisms, which significantly decreases the false positive rate (Wright et al., 2013; Backofen et al., 2018). In order to maximize the reliability of our regulatory interactions, we selected the most dissimilar sRNA homologs from the same genus and from the same species predicted by GLASSgo with more than 80% of similarity for the sRNA interaction prediction with CopraRNA (Wright et al., 2013). This procedure increases the chances of our regulatory interactions to be true because they will be conserved on a genus- or species-level. This, along with the filtering of the fifteen best-ranked CopraRNA predictions with *p*-value < 0.01 makes our conservative predictions yielding strong candidates for hypothesis generation and future experimental assay design. Even though these predicted regulatory interactions can either activate or repress the mRNA expression, we provide no functional annotation for them.

By applying our GRN sRNA-enrichment pipeline, we identified TFs, sRNAs and sigma factors jointly forming regulatory circuits in the regulatory networks. We were able to identify feed forward loops, single input modules and dense overlapping regulons. With no information on TFs regulating sRNAs, feedback loops were not possible to be identified for these networks. Furthermore, we presented the occurrences in which the co-regulation by sRNAs and TFs were also observed in other studied organisms. We highlighted genes in regulatory circuits involved in the following pathways: methionine biosynthesis and metabolism of cofactors and vitamins in *C. jeikeium;* TCA cycle and carbohydrate metabolism in *C. diphtheriae*; and TCA cycle, phosphate metabolism and cell division in *C. glutamicum*.

In our gene ontology analysis, ATP-binding is the molecular process with the most amount of genes potentially regulated by sRNAs in all studied organisms. This is not surprising, given the immense importance of ATP for the survival, growth and replication of all living organisms. In bacteria, ATP is associated with virulence factors and can even regulate virulence genes, e.g., the *mgtC* gene in *Salmonella* (Klein and Lewinson, 2011; Lee and Groisman, 2012; Mempin et al., 2013). Besides that, the other molecular processes with which most genes are associated are DNA binding and Metal ion binding, showing a probable strong influence of sRNA in these molecular functions. In *C. diphtheria* NCTC 13129, the sRNA Cd-NCTC13129-sRNA-2 potentially regulates the transcription factor *ydfH*, which regulates the global regulator *glxR*. Additionally, it is the regulator with the largest amount of regulations known in the *Corynebacterium* species. Likewise, in *C. jeikeium*, the sRNA scjk1464.1 regulates the transcription factors *dtxR* and *mcbR*. *DtxR* is the master regulator of iron metabolism in *C. glutamicum* (Wennerhold and Bott, 2006; Schröder and Tauch, 2010) and the TetR family regulator *mcbR* is involved in biofilm formation in *E. coli* (Zhang et al., 2008). Note that in *C. glutamicum* cg0350 (*glxR* homolog) is potentially regulated by the sRNA cgb_1195 and forms a feed forward loop together with this sRNA, *sdhCD*, and *acn*.

Amongst the genes potentially regulated by sRNAs, note the virulence factor *fagC* in *C. pseudotuberculosis*, the candidate virulence factor *rpfI* in *C. ulcerans* and the potential drug target *rpsH* in *C. diphtheriae*. We also observed the heat shock protein GroEL and the histidine kinase TcsS4 being regulated by sRNAs in *C. jeikeium*. While heat shock proteins are essential for bacterial survival and were recently associated with virulence and drug resistance (Neckers and Tatu, 2008), two-component systems are known as regulators of virulence factors and genes related to adhesion, pilus formation and drug resistance (López-Gońi et al., 2002; Matsushita and Janda, 2002; Tiwari et al., 2014). Moreover, the genes related to survival and adaptation in the *nif* operon and in the *suf* gene cluster (Stock et al., 1989; Huet et al., 2005) are regulated by the same sRNA and transcription factor in *C. jeikeium*. Genes of biotechnological interest, such as *pfkA in C. pseudotuberculosis, rhtC* in *C. jeikeium*, and *pyk* in *C. diphtheriae*, were also pointed out as sRNA targets. These genes are associated with L-threonine production, L-serine fermentation and lactic acid production in *C. glutamicum*, respectively. These molecules are largely used in the food industry (Diesveld et al., 2009; Chai et al., 2016; Zhang X. et al., 2017). The presented regulations show the potential of sRNAs to regulate genes of medical, veterinary and biotechnological interest in corynebacterial species.

## Conclusion

We introduce the sRNA regulatory networks integrated with the transcriptional gene regulatory networks of *C. glutamicum*, *C. pseudotuberculosis*, *C. ulcerans*, *C. diphtheriae*, *C. jeikeium*, and *C. efficiens*. This integration allowed us to identify sRNAs and TFs forming generalizable patterns, such as feed forward loops, dense overlapping regulons and single-input modules. It indicates sRNAs and TFs jointly orchestrating the regulation of corynebacterial gene expression, suggesting that sRNAs may have a great impact in modeling the gene expression of important biological processes in corynebacteria. Our results suggest several genes for further experimental investigation in the studied organisms. Amongst them, note the potential regulation of *mraZ*, which is conserved in four organisms of this study, and of the virulence factor *fagC*, which is potentially regulated by *dtxR* and one sRNA in both *C. pseudotuberculosis* and *C. ulcerans*. We believe that with CoryneRegNet 7.5, in which we implemented the integrated networks with extended visualization and querying functionality, we move an additional step toward understanding the corynebacterial regulatory mechanisms and provide new starting points to guide future experimental assays to comprehend the regulatory mechanisms underlying pathogenicity, survival, adaptation and amino acid production in the *Corynebacterium* genus.

## Data Availability Statement

The datasets presented in this study can be found in online repositories. The names of the repository/repositories and accession number(s) can be found below: https://www.exbio.wzw.tum.de/coryneregnet/processToDownalod.htm.

## Author Contributions

MP, MR, RB, VA, and JB conceptualized this work. MP and DP developed the software and wrote the manuscript. MP performed the analysis. VA, RK, and JB supervised the work. MR, RB, RK, FA, AP, VA, and JB reviewed the manuscript. All authors contributed to the article and approved the submitted version.

## Conflict of Interest

The authors declare that the research was conducted in the absence of any commercial or financial relationships that could be construed as a potential conflict of interest.

## References

[B1] Ahmed W. Hafeez M. A. Mahmood S. (2018). Identification and functional characterization of bacterial small non-coding RNAs and their target: a review. *Gene Rep.* 10 167–176. 10.1016/j.genrep.2018.01.001

[B2] Altuvia S. (2007). Identification of bacterial small non-coding RNAs: experimental approaches. *Curr. Opin. Microbiol.* 10 257–261. 10.1016/j.mib.2007.05.003 17553733

[B3] Araujo F. A. Barh D. Silva A. Guimarães L. Ramos R. T. J. (2018). GO FEAT: a rapid web-based functional annotation tool for genomic and transcriptomic data. *Sci. Rep.* 8:1794. 10.1038/s41598-018-20211-9 29379090 PMC5789007

[B4] Arrieta-Ortiz M. L. Hafemeister C. Shuster B. Baliga N. S. Bonneau R. Eichenberger P. (2020). Inference of bacterial small RNA regulatory networks and integration with transcription factor-driven regulatory networks. *mSystems* 5 e00057-20. 10.1128/mSystems.00057-20 32487739 PMC8534726

[B5] Backofen R. Gorodkin J. Hofacker I. L. Stadler P. F. (2018). Comparative RNA genomics. *Methods Mol. Biol*. 1704 363–400. 10.1007/978-1-4939-7463-4_14 29277874

[B6] Barquist L. Vogel J. (2015). Accelerating discovery and functional analysis of small RNAs with new technologies. *Annu. Rev. Genet.* 49 367–394. 10.1146/annurev-genet-112414-054804 26473381

[B7] Beisel C. L. Storz G. (2010). Base pairing small RNAs and their roles in global regulatory networks. *FEMS Microbiol. Rev.* 34 866–882. 10.1111/j.1574-6976.2010.00241.x 20662934 PMC2920360

[B8] Bernhart S. H. Hofacker I. L. Will S. Gruber A. R. Stadler P. F. (2008). RNAalifold: improved consensus structure prediction for RNA alignments. *BMC Bioinformatics* 9:474. 10.1186/1471-2105-9-474 19014431 PMC2621365

[B9] Billington S. J. Esmay P. A. Songer J. G. Jost B. H. (2002). Identification and role in virulence of putative iron acquisition genes from *Corynebacterium pseudotuberculosis*. *FEMS Microbiol. Lett.* 208 41–45. 10.1111/j.1574-6968.2002.tb11058.x 11934492

[B10] Bott M. (2007). Offering surprises: TCA cycle regulation in *Corynebacterium glutamicum*. *Trends Microbiol.* 15 417–425. 10.1016/j.tim.2007.08.004 17764950

[B11] Brosse A. Guillier M. (2018). Bacterial small RNAs in mixed regulatory networks. *Microbiol. Spectr.* 6 453–469. 10.1128/microbiolspec.RWR-0014-2017 29916348 PMC11633589

[B12] Bussmann M. Emer D. Hasenbein S. Degraf S. Eikmanns B. J. Bott M. (2009). Transcriptional control of the succinate dehydrogenase operon sdhCAB of *Corynebacterium glutamicum* by the cAMP-dependent regulator GlxR and the LuxR-type regulator RamA. *J. Biotechnol.* 143 173–182. 10.1016/j.jbiotec.2009.06.025 19583988

[B13] Chai X. Shang X. Zhang Y. Liu S. Liang Y. Zhang Y. (2016). A novel pyruvate kinase and its application in lactic acid production under oxygen deprivation in *Corynebacterium glutamicum*. *BMC Biotechnol.* 16:79. 10.1186/s12896-016-0313-6 27852252 PMC5112673

[B14] Chen C.-C. Qian X. Yoon B.-J. (2019). RNAdetect: efficient computational detection of novel non-coding RNAs. *Bioinformatics* 35 1133–1141. 10.1093/bioinformatics/bty765 30169792

[B15] De Lay N. Schu D. J. Gottesman S. (2013). Bacterial small RNA-based negative regulation: Hfq and its accomplices. *J. Biol. Chem.* 288 7996–8003. 10.1074/jbc.R112.441386 23362267 PMC3605619

[B16] Diallo I. Provost P. (2020). RNA-sequencing analyses of small bacterial RNAs and their emergence as virulence factors in host-pathogen interactions. *Int. J. Mol. Sci.* 21:1627. 10.3390/ijms21051627 32120885 PMC7084465

[B17] Diesveld R. Tietze N. Fürst O. Reth A. Bathe B. Sahm H. (2009). Activity of exporters of *Escherichia coli* in *Corynebacterium glutamicum*, and their use to increase L-threonine production. *J. Mol. Microbiol. Biotechnol.* 16 198–207. 10.1159/000142530 18594129

[B18] Draganova E. B. Akbas N. Adrian S. A. Lukat-Rodgers G. S. Collins D. P. Dawson J. H. (2015). Heme binding by I HmuT: function and heme environment. *Biochemistry* 54 6598–6609. 10.1021/acs.biochem.5b00666 26478504 PMC4943319

[B19] Eraso J. M. Markillie L. M. Mitchell H. D. Taylor R. C. Orr G. Margolin W. (2014). The highly conserved MraZ protein is a transcriptional regulator in *Escherichia coli*. *J. Bacteriol.* 196 2053–2066. 10.1128/JB.01370-13 24659771 PMC4010979

[B20] Faner M. A. Feig A. L. (2013). Identifying and characterizing Hfq-RNA interactions. *Methods* 63 144–159. 10.1016/j.ymeth.2013.04.023 23707622 PMC3787079

[B21] Frazzon J. (2003). Formation of iron–sulfur clusters in bacteria: an emerging field in bioinorganic chemistry. *Curr. Opin. Chem. Biol.* 7 166–173. 10.1016/s1367-5931(03)00021-8 12714048

[B22] Freyhult E. K. Bollback J. P. Gardner P. P. (2007). Exploring genomic dark matter: a critical assessment of the performance of homology search methods on noncoding RNA. *Genome Res.* 17 117–125. 10.1101/gr.5890907 17151342 PMC1716261

[B23] Freyre-González J. A. Tauch A. (2017). Functional architecture and global properties of the *Corynebacterium glutamicum* regulatory network: novel insights from a dataset with a high genomic coverage. *J. Biotechnol.* 257 199–210. 10.1016/j.jbiotec.2016.10.025 27829123

[B24] Georg J. Lalaouna D. Hou S. Lott S. C. Caldelari I. Marzi S. (2020). The power of cooperation: experimental and computational approaches in the functional characterization of bacterial sRNAs. *Mol. Microbiol.* 113 603–612. 10.1111/mmi.14420 31705780 PMC7154689

[B25] Gomide A. C. P. de Sá P. G. Cavalcante A. L. Q. de Jesus Sousa T. Gomes L. G. R. Ramos R. T. J. (2018). Heat shock stress: profile of differential expression in *Corynebacterium pseudotuberculosis* biovar Equi. *Gene* 645 124–130. 10.1016/j.gene.2017.12.015 29246537

[B26] Gripenland J. Netterling S. Loh E. Tiensuu T. Toledo-Arana A. Johansson J. (2010). RNAs: regulators of bacterial virulence. *Nat. Rev. Microbiol.* 8 857–866. 10.1038/nrmicro2457 21079634

[B27] Gruber A. R. Findeiß S. Washietl S. Hofacker I. L. Stadler P. F. (2010). RNAz 2.0: improved noncoding RNA detection. *Pac. Symp. Biocomput.* 69–79. 19908359

[B28] Han K. Tjaden B. Lory S. (2016). GRIL-seq provides a method for identifying direct targets of bacterial small regulatory RNA by in vivo proximity ligation. *Nat. Microbiol.* 2:16239. 10.1038/nmicrobiol.2016.239 28005055 PMC5567838

[B29] Hanawa T. Yamanishi S. Murayama S. Yamamoto T. Kamiya S. (2002). Participation of DnaK in expression of genes involved in virulence of Listeria monocytogenes. *FEMS Microbiol. Lett.* 214 69–75. 10.1111/j.1574-6968.2002.tb11326.x 12204374

[B30] Hartmann M. Barsch A. Niehaus K. Pühler A. Tauch A. Kalinowski J. (2004). The glycosylated cell surface protein Rpf2, containing a resuscitation-promoting factor motif, is involved in intercellular communication of *Corynebacterium glutamicum*. *Arch. Microbiol.* 182 299–312. 10.1007/s00203-004-0713-1 15480574

[B31] Hassan S. S. Jamal S. B. Radusky L. G. Tiwari S. Ullah A. Ali J. (2018). The druggable pocketome of *Corynebacterium diphtheriae*: a new approach for in silico putative druggable targets. *Front. Genet.* 9:44. 10.3389/fgene.2018.00044 29487617 PMC5816920

[B32] Huang H.-Y. Chang H.-Y. Chou C.-H. Tseng C.-P. Ho S.-Y. Yang C.-D. (2009). sRNAMap: genomic maps for small non-coding RNAs, their regulators and their targets in microbial genomes. *Nucleic Acids Res.* 37 D150–D154. 10.1093/nar/gkn852 19015153 PMC2686527

[B33] Huang M. Zhao Y. Feng L. Zhu L. Zhan L. Chen X. (2020). Role of the ClpX from *Corynebacterium crenatum* involved in stress responses and energy metabolism. *Appl. Microbiol. Biotechnol.* 104 5505–5517. 10.1007/s00253-020-10597-w 32300856

[B34] Huet G. Daffeì M. Saves I. (2005). Identification of the *Mycobacterium tuberculosis* SUF machinery as the exclusive mycobacterial system of [Fe-S] cluster assembly: evidence for its implication in the pathogen’s survival. *J. Bacteriol.* 187 6137–6146. 10.1128/jb.187.17.6137-6146.2005 16109955 PMC1196142

[B35] Ishige T. Krause M. Bott M. Wendisch V. F. Sahm H. (2003). The phosphate starvation stimulon of *Corynebacterium glutamicum* determined by DNA microarray analyses. *J. Bacteriol.* 185 4519–4529. 10.1128/jb.185.15.4519-4529.2003 12867461 PMC165763

[B36] Jamal S. B. Hassan S. S. Tiwari S. Viana M. V. de Jesus Benevides L. Ullah A. (2017). An integrative in-silico approach for therapeutic target identification in the human pathogen *Corynebacterium diphtheriae*. *PLoS One* 12:e0186401. 10.1371/journal.pone.0186401 29049350 PMC5648181

[B37] Jochmann N. Kurze A.-K. Czaja L. F. Brinkrolf K. Brune I. Hüser A. T. (2009). Genetic makeup of the *Corynebacterium glutamicum* LexA regulon deduced from comparative transcriptomics and *in vitro* DNA band shift assays. *Microbiology* 155, 1459–1477. 10.1099/mic.0.025841-0 19372162

[B38] Kalvari I. Argasinska J. Quinones-Olvera N. Nawrocki E. P. Rivas E. Eddy S. R. (2018). Rfam 13.0: shifting to a genome-centric resource for non-coding RNA families. *Nucleic Acids Res.* 46 D335–D342. 10.1093/nar/gkx1038 29112718 PMC5753348

[B39] Kalvari I. Nawrocki E. P. Ontiveros-Palacios N. Argasinska J. Lamkiewicz K. Marz M. (2021). Rfam 14: expanded coverage of metagenomic, viral and microRNA families. *Nucleic Acids Res.* 49 D192–D200. 10.1093/nar/gkaa1047 33211869 PMC7779021

[B40] Klein J. S. Lewinson O. (2011). Bacterial ATP-driven transporters of transition metals: physiological roles, mechanisms of action, and roles in bacterial virulence. *Metallomics* 3 1098–1108. 10.1039/c1mt00073j 21901186

[B41] Lalaouna D. Massé E. (2015). Identification of sRNA interacting with a transcript of interest using MS2-affinity purification coupled with RNA sequencing (MAPS) technology. *Genom. Data* 5 136–138. 10.1016/j.gdata.2015.05.033 26484242 PMC4583644

[B42] Larsen S. J. Röttger R. Schmidt H. H. H. W. Baumbach J. (2019). E. coli gene regulatory networks are inconsistent with gene expression data. *Nucleic Acids Res.* 47 85–92. 10.1093/nar/gky1176 30462289 PMC6326786

[B43] Lee E.-J. Groisman E. A. (2012). Control of a *Salmonella* virulence locus by an ATP-sensing leader messenger RNA. *Nature* 486 271–275. 10.1038/nature11090 22699622 PMC3711680

[B44] Lee H.-J. Gottesman S. (2016). sRNA roles in regulating transcriptional regulators: Lrp and SoxS regulation by sRNAs. *Nucleic Acids Res.* 44 6907–6923. 10.1093/nar/gkw358 27137887 PMC5001588

[B45] Li L. Huang D. Cheung M. K. Nong W. Huang Q. Kwan H. S. (2013). BSRD: a repository for bacterial small regulatory RNA. *Nucleic Acids Res.* 41 D233–D238. 10.1093/nar/gks1264 23203879 PMC3531160

[B46] Lin Y. Liu T. Cui T. Wang Z. Zhang Y. Tan P. (2020). RNAInter in 2020: RNA interactome repository with increased coverage and annotation. *Nucleic Acids Res.* 48 D189–D197. 10.1093/nar/gkz804 31906603 PMC6943043

[B47] López-Gońi I. Guzmán-Verri C. Manterola L. Sola-Landa A. Moriyón I. Moreno E. (2002). Regulation of *Brucella* virulence by the two-component system BvrR/BvrS. *Vet. Microbiol.* 90 329–339. 10.1016/S0378-1135(02)00218-3 12414153

[B48] Lott S. C. Schäfer R. A. Mann M. Backofen R. Hess W. R. Voß B. (2018). GLASSgo – automated and reliable detection of sRNA homologs from a single input sequence. *Front. Genet.* 9:124. 10.3389/fgene.2018.00124 29719549 PMC5913331

[B49] Maeda T. Tanaka Y. Takemoto N. Hamamoto N. Inui M. (2016). RNase III mediated cleavage of the coding region of mraZ mRNA is required for efficient cell division in *Corynebacterium glutamicum*. *Mol. Microbiol.* 99 1149–1166. 10.1111/mmi.13295 26713407

[B50] Mandin P. Chareyre S. Barras F. (2016). A regulatory circuit composed of a transcription factor, IscR, and a regulatory RNA, RyhB, controls Fe-S cluster delivery. *mBio* 7:e00966-16. 10.1128/mbio.00966-16 27651365 PMC5040110

[B51] Massé E. Vanderpool C. K. Gottesman S. (2005). Effect of RyhB small RNA on global iron use in *Escherichia coli*. *J. Bacteriol.* 187 6962–6971. 10.1128/JB.187.20.6962-6971.2005 16199566 PMC1251601

[B52] Matsushita M. Janda K. D. (2002). Histidine kinases as targets for new antimicrobial agents. *Bioorg. Med. Chem.* 10 855–867. 10.1016/s0968-0896(01)00355-8 11836091

[B53] Melamed S. Peer A. Faigenbaum-Romm R. Gatt Y. E. Reiss N. Bar A. (2016). Global mapping of small RNA-target interactions in bacteria. *Mol. Cell* 63 884–897. 10.1016/j.molcel.2016.07.026 27588604 PMC5145812

[B54] Mempin R. Tran H. Chen C. Gong H. Kim Ho K. Lu S. (2013). Release of extracellular ATP by bacteria during growth. *BMC Microbiol.* 13:301. 10.1186/1471-2180-13-301 24364860 PMC3882102

[B55] Mentz A. Neshat A. Pfeifer-Sancar K. Pühler A. Rückert C. Kalinowski J. (2013). Comprehensive discovery and characterization of small RNAs in *Corynebacterium glutamicum* ATCC 13032. *BMC Genomics* 14:714. 10.1186/1471-2164-14-714 24138339 PMC4046766

[B56] Nawrocki E. P. Eddy S. R. (2013). Infernal 1.1: 100-fold faster RNA homology searches. *Bioinformatics* 29 2933–2935. 10.1093/bioinformatics/btt509 24008419 PMC3810854

[B57] Neckers L. Tatu U. (2008). Molecular chaperones in pathogen virulence: emerging new targets for therapy. *Cell Host Microbe* 4 519–527. 10.1016/j.chom.2008.10.011 19064253 PMC2752846

[B58] Nitzan M. Rehani R. Margalit H. (2017). Integration of bacterial small RNAs in regulatory networks. *Annu. Rev. Biophys.* 46 131–148. 10.1146/annurev-biophys-070816-034058 28532217

[B59] Oguiza J. A. Marcos A. T. Malumbres M. Martín J. F. (1996). Multiple sigma factor genes in *Brevibacterium lactofermentum*: characterization of sigA and sigB. *J. Bacteriol.* 178 550–553. 10.1128/jb.178.2.550-553.1996 8550480 PMC177692

[B60] Outten F. W. Wayne Outten F. (2015). Recent advances in the Suf Fe–S cluster biogenesis pathway: beyond the Proteobacteria. *Biochim. Biophys. Acta Mol. Cell Res.* 1853 1464–1469. 10.1016/j.bbamcr.2014.11.001 25447545 PMC4390423

[B61] Pain A. Ott A. Amine H. Rochat T. Bouloc P. Gautheret D. (2015). An assessment of bacterial small RNA target prediction programs. *RNA Biol.* 12 509–513. 10.1080/15476286.2015.1020269 25760244 PMC4615726

[B62] Pao G. M. Saier M. H. Jr. (1995). Response regulators of bacterial signal transduction systems: selective domain shuffling during evolution. *J. Mol. Evol.* 40 136–154. 10.1007/BF00167109 7699720

[B63] Papenfort K. Vanderpool C. K. (2015). Target activation by regulatory RNAs in bacteria. *FEMS Microbiol. Rev.* 39 362–378. 10.1093/femsre/fuv016 25934124 PMC4542691

[B64] Parise D. Parise M. T. D. Kataka E. Kato R. B. List M. Tauch A. (2021). On the consistency between gene expression and the gene regulatory network of *Corynebacterium glutamicum*. *Netw. Syst. Med.* 4 51–59. 10.1089/nsm.2020.0014 33796877 PMC8006670

[B65] Parise M. T. D. Parise D. Kato R. B. Pauling J. K. Tauch A. Azevedo V. A. (2020). CoryneRegNet 7, the reference database and analysis platform for corynebacterial gene regulatory networks. *Sci. Data* 7:142. 10.1038/s41597-020-0484-9 32393779 PMC7214426

[B66] Pischimarov J. Kuenne C. Billion A. Hemberger J. Cemič F. Chakraborty T. (2012). sRNAdb: a small non-coding RNA database for gram-positive bacteria. *BMC Genomics* 13:384. 10.1186/1471-2164-13-384 22883983 PMC3439263

[B67] Pogson C. A. Simmons C. P. Strugnell R. A. Hodgson A. L. (1996). Cloning and manipulation of the *Corynebacterium pseudotuberculosis recA* gene for live vaccine vector development. *FEMS Microbiol. Lett.* 142, 139–145. 10.1111/j.1574-6968.1996.tb08421.x 8810496

[B68] Polen T. Schluesener D. Poetsch A. Bott M. Wendisch V. F. (2007). Characterization of citrate utilization in *Corynebacterium glutamicum* by transcriptome and proteome analysis. *FEMS Microbiol. Lett.* 273 109–119. 10.1111/j.1574-6968.2007.00793.x 17559405

[B69] Resende B. C. Rebelato A. B. D’Afonseca V. Santos A. R. Stutzman T. Azevedo V. A. (2011). DNA repair in *Corynebacterium* model. *Gene* 482, 1–7. 10.1016/j.gene.2011.03.008 21497183

[B70] Rinke T. F. Bekelie S. Osland A. Miko T. L. Hermans P. W. M. Soolingen D. (1992). Mycobacteria contain two groEL genes: the second *Mycobacterium leprae* groEL gene is arranged in an operon with groES. *Mol. Microbiol.* 6 1995–2007. 10.1111/j.1365-2958.1992.tb01372.x 1354834

[B71] Rückert C. Pühler A. Kalinowski J. (2003). Genome-wide analysis of the L-methionine biosynthetic pathway in *Corynebacterium glutamicum* by targeted gene deletion and homologous complementation. *J. Biotechnol.* 104 213–228. 10.1016/s0168-1656(03)00158-5 12948640

[B72] Santos-Zavaleta A. Salgado H. Gama-Castro S. Sánchez-Pérez M. Gómez-Romero L. Ledezma-Tejeida D. (2019). RegulonDB v 10.5: tackling challenges to unify classic and high throughput knowledge of gene regulation in E. coli K-12. *Nucleic Acids Res.* 47 D212–D220. 10.1093/nar/gky1077 30395280 PMC6324031

[B73] Schröder J. Tauch A. (2010). Transcriptional regulation of gene expression in *Corynebacterium glutamicum*: the role of global, master and local regulators in the modular and hierarchical gene regulatory network. *FEMS Microbiol. Rev.* 34 685–737. 10.1111/j.1574-6976.2010.00228.x 20491930

[B74] Sharma C. M. Vogel J. (2009). Experimental approaches for the discovery and characterization of regulatory small RNA. *Curr. Opin. Microbiol.* 12 536–546. 10.1016/j.mib.2009.07.006 19758836

[B75] Sievers F. Wilm A. Dineen D. Gibson T. J. Karplus K. Li W. (2011). Fast, scalable generation of high-quality protein multiple sequence alignments using Clustal Omega. *Mol. Syst. Biol.* 7:539. 10.1038/msb.2011.75 21988835 PMC3261699

[B76] Sittka A. Lucchini S. Papenfort K. Sharma C. M. Rolle K. Binnewies T. T. (2008). Deep sequencing analysis of small noncoding RNA and mRNA targets of the global post-transcriptional regulator, Hfq. *PLoS Genet.* 4:e1000163. 10.1371/journal.pgen.1000163 18725932 PMC2515195

[B77] Stats (2020). *p.adjust.* Available online at: https://www.rdocumentation.org/packages/stats/versions/3.6.2/topics/p.adjust (accessed August 16, 2020)

[B78] Stock J. B. Ninfa A. J. Stock A. M. (1989). Protein phosphorylation and regulation of adaptive responses in bacteria. *Microbiol. Rev.* 53 450–490. 10.1128/mmbr.53.4.450-490.1989 2556636 PMC372749

[B79] The RNAcentral Consortium, (2019). RNAcentral: a hub of information for non-coding RNA sequences. *Nucleic Acids Res.* 47 D1250–D1251. 10.1093/nar/gky1206 30535383 PMC6323998

[B80] Tiwari S. da Costa M. P. Almeida S. Hassan S. S. Jamal S. B. Oliveira A. (2014). C. pseudotuberculosis Phop confers virulence and may be targeted by natural compounds. *Integr. Biol.* 6 1088–1099. 10.1039/c4ib00140k 25212181

[B81] Trost E. Al-Dilaimi A. Papavasiliou P. Schneider J. Viehoever P. Burkovski A. (2011). Comparative analysis of two complete *Corynebacterium ulcerans* genomes and detection of candidate virulence factors. *BMC Genomics* 12:383. 10.1186/1471-2164-12-383 21801446 PMC3164646

[B82] Wang J. Liu T. Zhao B. Lu Q. Wang Z. Cao Y. (2016). sRNATarBase 3.0: an updated database for sRNA-target interactions in bacteria. *Nucleic Acids Res.* 44 D248–D253. 10.1093/nar/gkv1127 26503244 PMC4702819

[B83] Waters L. S. Storz G. (2009). Regulatory RNAs in bacteria. *Cell* 136 615–628. 10.1016/j.cell.2009.01.043 19239884 PMC3132550

[B84] Waters S. A. McAteer S. P. Kudla G. Pang I. Deshpande N. P. Amos T. G. (2017). Small RNA interactome of pathogenic E. coli revealed through crosslinking of RN ase E. *EMBO J.* 36 374–387. 10.15252/embj.201694639 27836995 PMC5286369

[B85] Wennerhold J. Bott M. (2006). The DtxR regulon of *Corynebacterium glutamicum*. *J. Bacteriol.* 188 2907–2918. 10.1128/JB.188.8.2907-2918.2006 16585752 PMC1446976

[B86] Wright P. R. Georg J. (2018). Workflow for a computational analysis of an sRNA candidate in bacteria. *Methods Mol. Biol.* 1737 3–30. 10.1007/978-1-4939-7634-8_1 29484584

[B87] Wright P. R. Richter A. S. Papenfort K. Mann M. Vogel J. Hess W. R. (2013). Comparative genomics boosts target prediction for bacterial small RNAs. *Proc. Natl. Acad. Sci. U.S.A.* 110 E3487–E3496. 10.1073/pnas.1303248110 23980183 PMC3773804

[B88] Wu T. P. Ruan K. C. Liu W. Y. (1996). A fluorescence-labeling method for sequencing small RNA on polyacrylamide gel. *Nucleic Acids Res.* 24 3472–3473. 10.1093/nar/24.17.3472 8811106 PMC146087

[B89] Yoon J. Woo H. M. (2018). CRISPR interference-mediated metabolic engineering of *Corynebacterium glutamicum* for homo-butyrate production. *Biotechnol. Bioeng.* 115 2067–2074. 10.1002/bit.26720 29704438

[B90] Zhang X. Yao L. Xu G. Zhu J. Zhang X. Shi J. (2017). Enhancement of fructose utilization from sucrose in the cell for improved l-serine production in engineered *Corynebacterium glutamicum*. *Biochem. Eng. J.* 118 113–122. 10.1016/j.bej.2016.11.021

[B91] Zhang X.-S. García-Contreras R. Wood T. K. (2008). *Escherichia coli* transcription factor YncC (McbR) regulates colanic acid and biofilm formation by repressing expression of periplasmic protein YbiM (McbA). *ISME J.* 2 615–631. 10.1038/ismej.2008.24 18309357

[B92] Zhang Y. Huang H. Zhang D. Qiu J. Yang J. Wang K. (2017). A review on recent computational methods for predicting noncoding RNAs. *BioMed. Res. Int.* 2017:9139504. 10.1155/2017/9139504 28553651 PMC5434267

