# Supplementary material for: An Integrated Database of Small RNAs and Their Interplay With Transcriptional Gene Regulatory Networks in Corynebacteria
Source: Front Microbiol. 2021 Jun 17;12:656435. doi: 10.3389/fmicb.2021.656435 (PMC8247434; doi:10.3389/fmicb.2021.656435)
Supplement: Supplementary file 1 [file Data_Sheet_1.pdf]

Supplementary material

An integrated database of small RNAs and their interplay with transcriptional gene regulatory networks in corynebacteria

Mariana Teixeira Dornelles Parise[1,2], Doglas Parise[1,2], Flavia Figueira Aburjaile[2], Anne Cybelle Pinto Gomide[2], Rodrigo Bentes Kato[2], Martin Raden[3], Rolf Backofen[3], Vasco Ariston de Carvalho Azevedo[2], Jan Baumbach[1,4,5]

[1] Chair of Experimental Bioinformatics, TUM School of Life Sciences, Technical University of Munich, Munich, Germany.
[2] Institute of Biological Sciences, Universidade Federal de Minas Gerais, Belo Horizonte, Minas Gerais, Brazil.
[3] Bioinformatics, Department of Computer Science, University of Freiburg, Freiburg, Germany
[4] Computational Biomedicine lab, Department of Mathematics and Computer Science, University of Southern Denmark, Odense, Germany
[5] Chair of Computational Systems Biology, University of Hamburg, Hamburg, Germany

I. Supplementary Figures

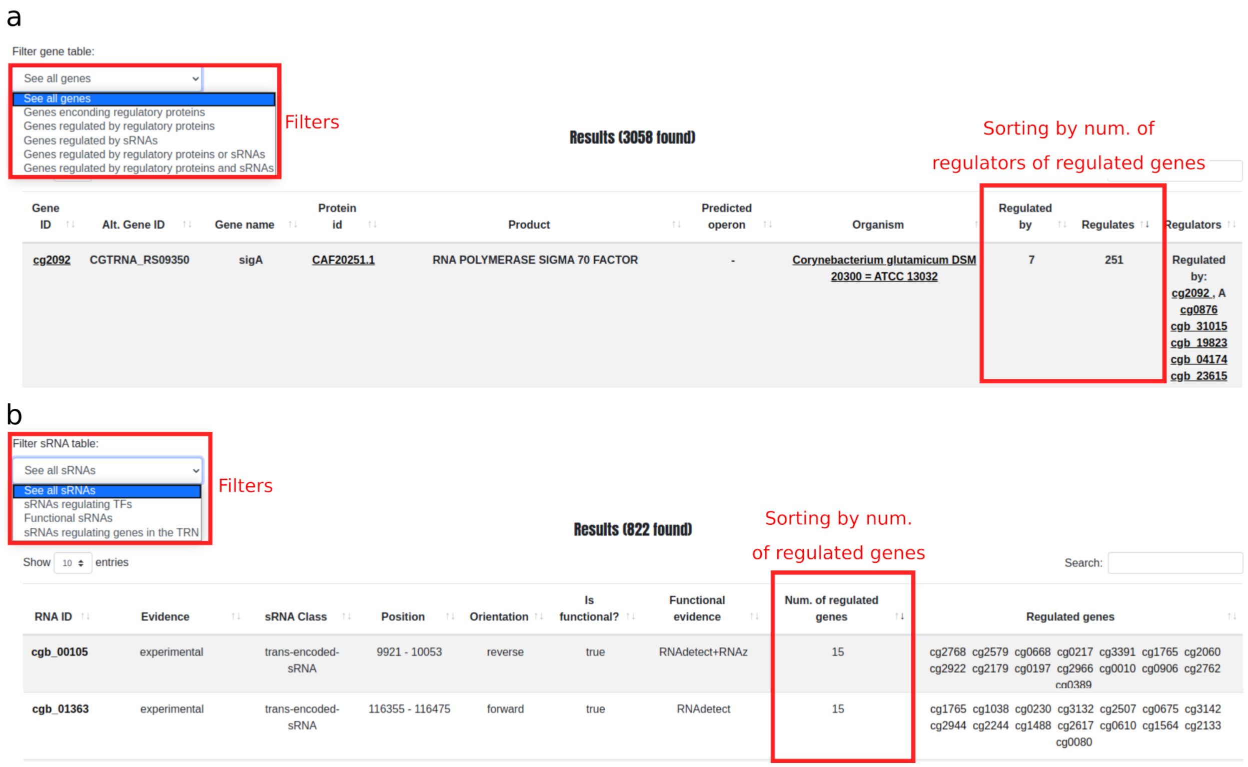

Supplementary Figure 1 - Table-oriented views.

a

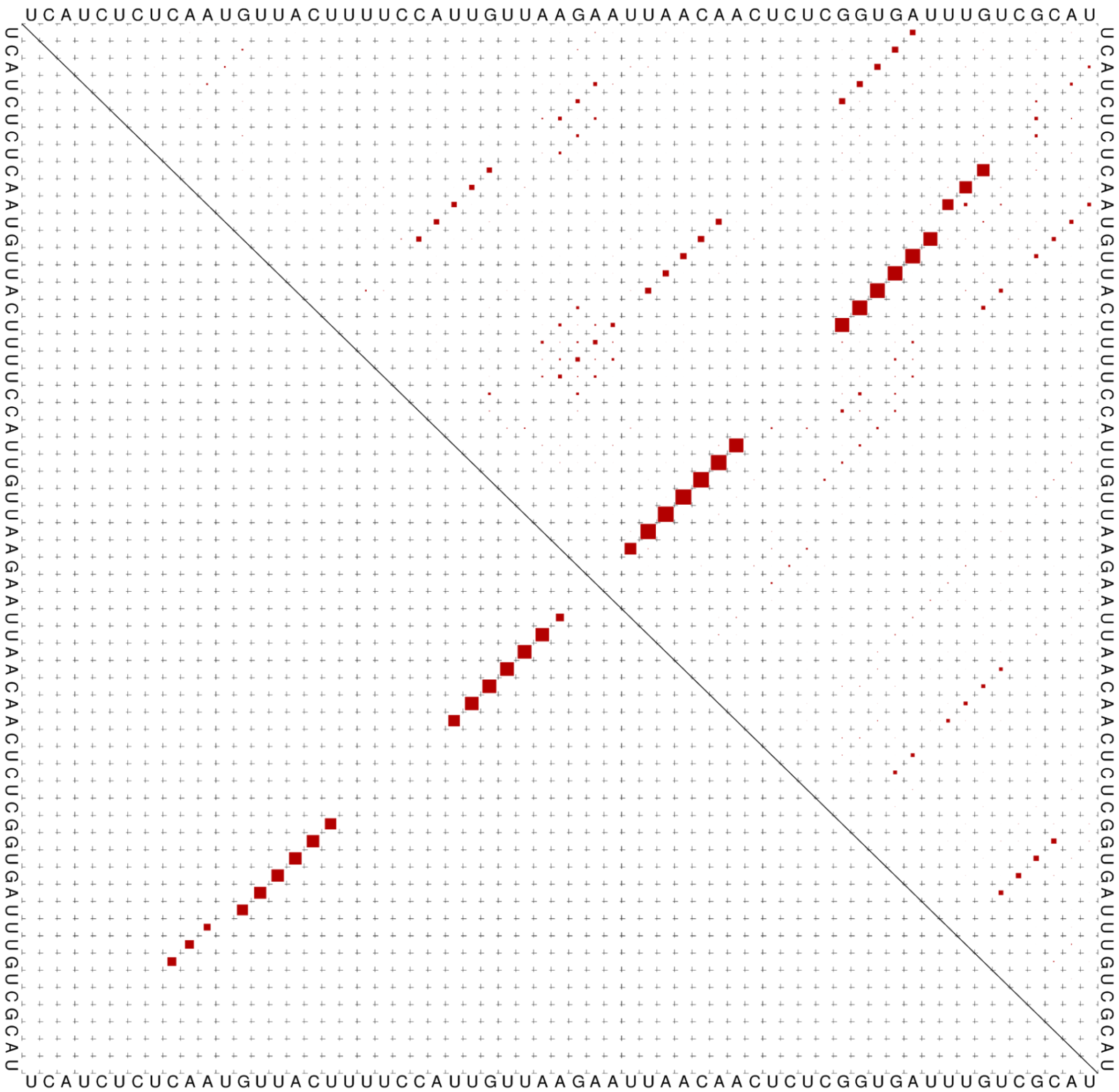

b

```
                ........(((.((((((......(((((((...))))))).....))))))))).....
NC_006958       TCATCTCTCAATGTTACTTTTCCATTGTTAAGAATTAACAACTCTCGGTGATTTGTCGCA 60
NZ_CP025534     TCATCTCTCAATGTTACTTTTCCATTGTTAAGAATTAACAACTCTCGGTGATTTGTCGCA 60
NZ_CP025533     TCATCTCTCAATGTTACTTTTCCATTGTTAAGAATTAACAACTCTCGGTGATTTGTCGCA 60
                .........10........20........30........40........50........6

                .
NC_006958       T 61
NZ_CP025534     T 61
NZ_CP025533     T 61
                0
```

Supplementary Figure 2 - Dot Plot (a) and plot of the homologue alignment (b) of the sRNA cgb_07555.

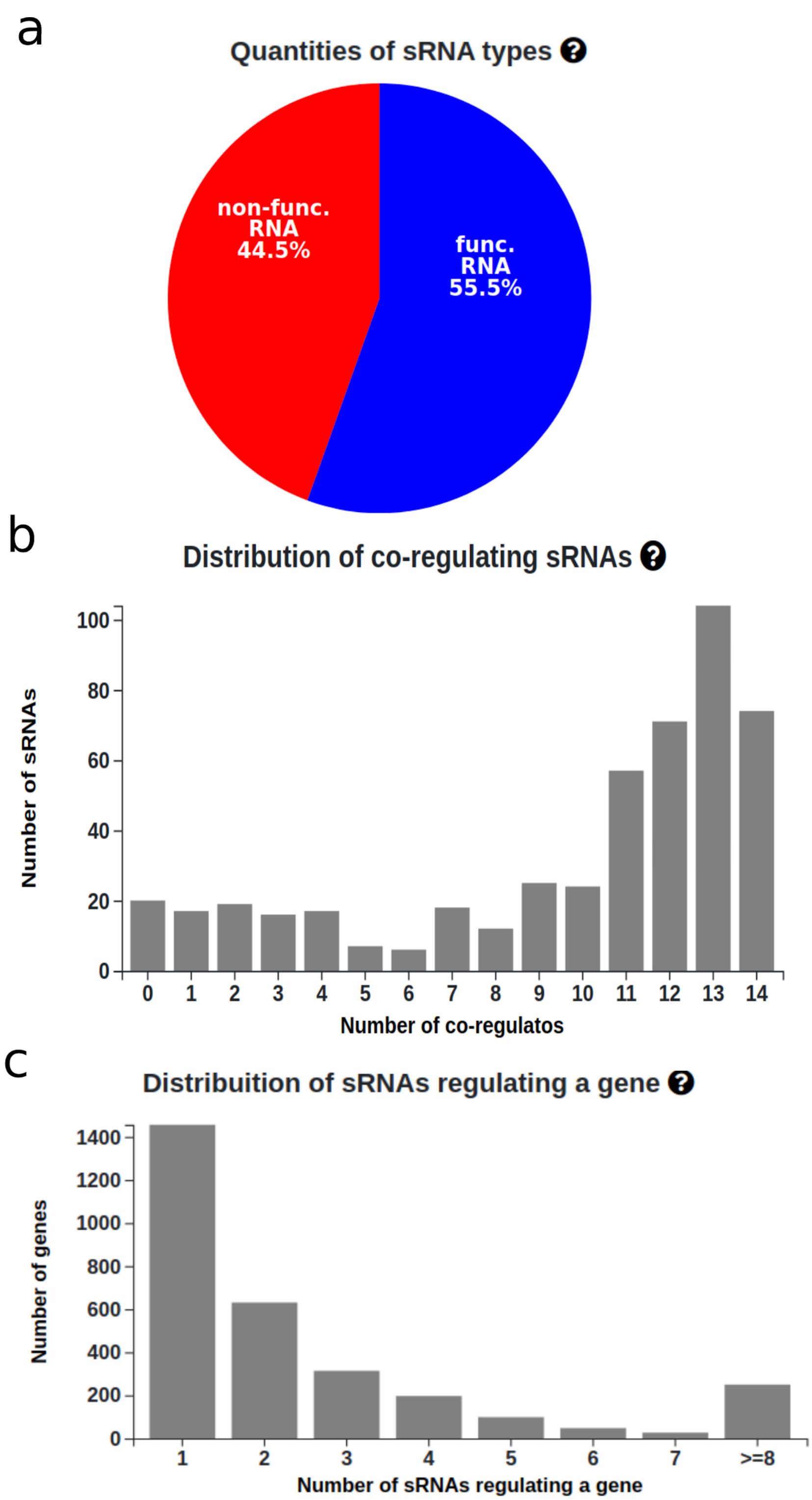

Supplementary Figure 3 - Novel graphs in sRNA statistics page. (a) Percentage of sRNA types, in red non-functional RNAs and in blue functional RNAs (b) Distribution of sRNAs co-regulating a gene (c) Distribution of sRNAs regulating a gene.

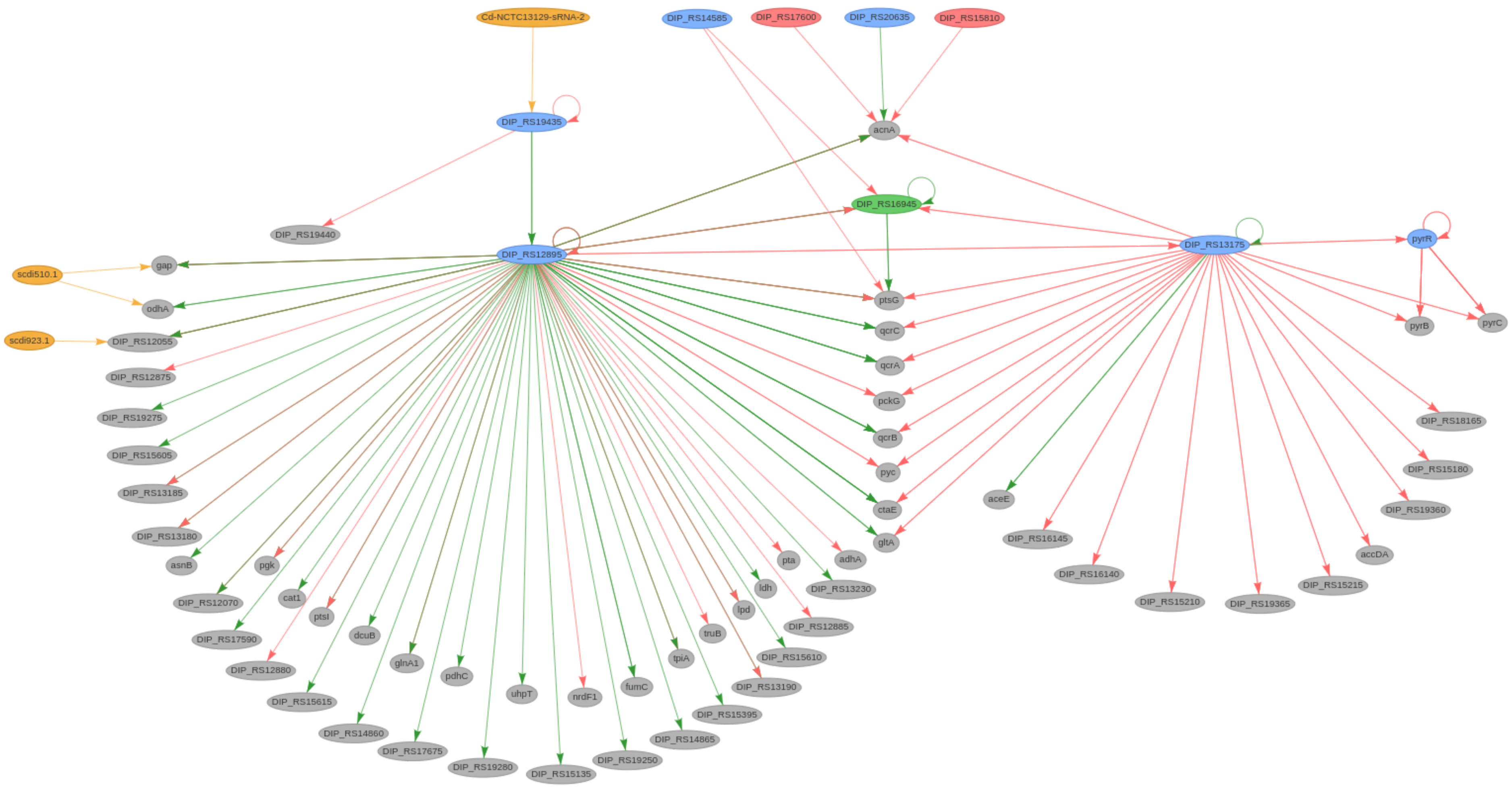

Supplementary Figure 4 - Complete set of genes predicted to be regulated by Cd-NCTC13129-sRNA-2. In the network, green nodes represent activator proteins, red nodes represent repressor proteins, blue nodes represent dual regulators, orange nodes represent sRNAs and grey nodes represent target genes. The arrows represent the regulatory interactions and their colors represent the same roles as the ones in the nodes.

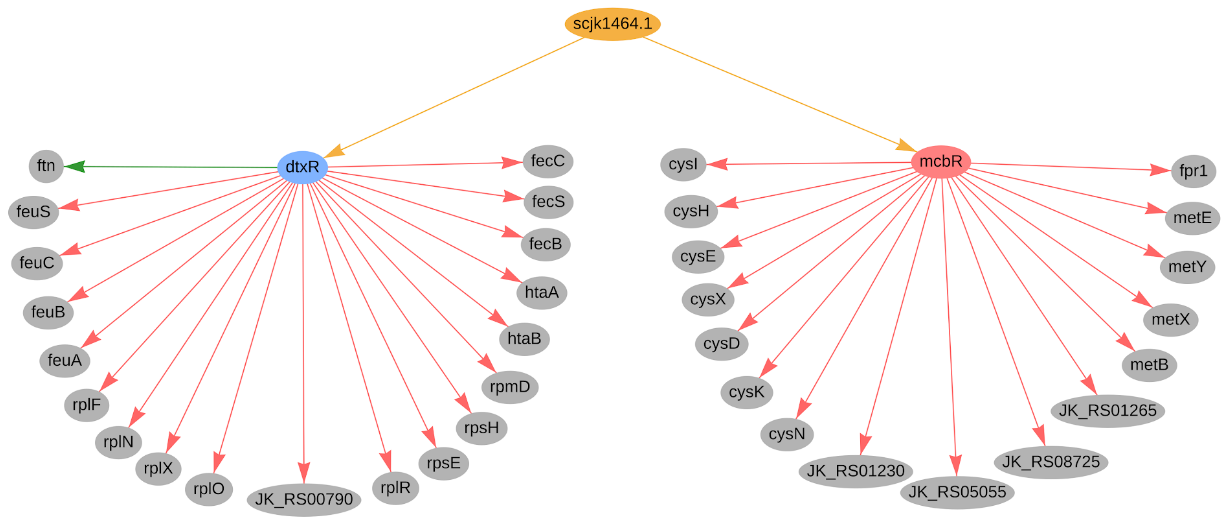

Supplementary Figure 5 - Regulatory network of the genes regulated by scjk1464.1 sRNA in *C. jeikeium*. In the network, green nodes represent activator proteins, red nodes represent repressor proteins, blue nodes represent dual regulators, orange nodes represent sRNAs and grey nodes represent target genes. The arrows represent the regulatory interactions and their colors represent the same roles as the ones in the nodes.

II. Detailed description on how to predict sRNAs and their regulatory networks for a bacteria of interest.

The first step to predict sRNAs and their regulatory interactions is to choose the bacteria of interest. In case you are looking for novel sRNAs in your bacteria of interest, the next step is to run cmsearch (Nawrocki and Eddy, 2013) with the nucleotide sequence of the genome of interest to identify possible sRNAs. To identify sRNAs regulatory interactions the following steps are performed.

```
$ cmsearch Rfam.cm bacteria_of_interest.fa
```

In the above command, 'Rfam.cm' is the Rfam database (http://ftp.ebi.ac.uk/pub/databases/Rfam/CURRENT/) and 'bacteria_of_interest.fa' is the nucleotide sequence of the genome of interest in fasta format. Further instructions on how to run cmsearch can be found in the work of Kalvari and collaborators (Kalvari et al., 2018).

In case you have a sRNA of interest and you want to verify if this sRNA is potentially present in your bacteria of interest, run GLASSgo (Lott et al., 2018) according to step one and verify whether it was predicted on your bacteria of interest or not.

We present the following analysis with an example srna named srna1:

```
>srna1
CGCACAGATCGATATCCCACTGCAAGAGCAGCTCATCGTCGAGTTCTACT
```

For each sRNA of interest:

1. The identification of sRNA regulatory interactions is based on their conservation in a species and genus level. To investigate this conservation, the first step is to run GLASSgo (Lott et al., 2018) for each one of the selected sRNAs.

   ```
   $ python3 GLASSgo.py -d ~/nt-database/nt -i srna1.fa -o output-srna1.fa
   ```

   In the above command, '~/nt-database/nt' is the path of the NCBI nucleotide database. The Nucleotide database is a collection of sequences from several sources, including GenBank, RefSeq, TPA and PDB. 'srna1.fa' is the nucleotide sequence of a sRNA.

2. Select the most distant homologues from the same species and from the same genus with ≥80% of similarity for every one of the selected sRNAs. In case no sRNA homologs of the same species fit this requirement, select two homologs sRNAs of different species in the same genus.

   To select the most distant sRNA homologous, we consider the most dissimilar homologous would be the most distant ones. To create the multi-fasta file, use RefSeq genome identifiers as homologs sRNA names, since those will be necessary for running CopraRNA (Wright et al., 2013).

   ```
   >REFSEQ-ID-SRNA1
   GAGAGATGCCCTCCTGGATGGGGAATACAGACCTTCGACAAGTCGTATTATCCCAGTTCAGAA
   GGGCGTTTCTCTTTTTCCCACGCCGCTACCCCTAAATCACATCGGTGTATCCAG
   >REFSEQ-ID-HOMOLOG-SRNA1
   GAGAGATGCCCTCCTGGATGGGGAATATAGACCTTCGACAAGTCGTATTATCCCAGTTCAGAA
   GGGTATTTCTCTTTTTCCCACGCCGCTACCCCAAAATCACATCGGTGGATCCAG
   >REFSEQ-ID-HOMOLOG-SRNA2
   GAGAGATGCCCTCCTGGATGGGGAATATAGACCTTCGACAAGTCGTATTATCCCAGTTCAGAA
   ```

GGGTATTTCTCTTTTTCCCACGCCGCTACCCCAAAATCACATCGGTGGATCCAG

3. Align the sequence of the selected sRNAs with the homologs selected on step 2 using clustalo (Sievers et al., 2011). The aligned sRNAs will be used as input for RNAz, RNAdetect and RNAalifold.

$ clustalo -i srna1-homologs.fa -o srna1-homologs.out --outfmt=clu

In the above command, 'srna1-homologs.fa' is the multifasta file with the three homolog sRNA and 'srna1-homologs.out' is the output of clustalo.

```
CLUSTAL O(1.2.4) multiple sequence alignment


REFSEQ-ID-SRNA1
GAGAGATGCCCTCCTGGATGGGGAATACAGACCTTCGACAAGTCGTATTATCCCAGTTCA
REFSEQ-ID-HOMOLOG-SRNA1
GAGAGATGCCCTCCTGGATGGGGAATATAGACCTTCGACAAGTCGTATTATCCCAGTTCA
REFSEQ-ID-HOMOLOG-SRNA2
GAGAGATGCCCTCCTGGATGGGGAATATAGACCTTCGACAAGTCGTATTATCCCAGTTCA
        *************************** ********************************

REFSEQ-ID-SRNA1
GAAGGGCGTTTCTCTTTTTCCCACGCCGCTACCCCTAAATCACATCGGTGTATCCAG
REFSEQ-ID-HOMOLOG-SRNA1
GAAGGGTATTTCTCTTTTTCCCACGCCGCTACCCCAAAATCACATCGGTGGATCCAG
REFSEQ-ID-HOMOLOG-SRNA2
GAAGGGTATTTCTCTTTTTCCCACGCCGCTACCCCAAAATCACATCGGTGGATCCAG
        ******  ************************** ************** ******
```

4. Run RNAz (Gruber et al., 2010) with the alignments obtained on step 3 in order to identify conserved functional structures.

$ RNAz srna1-homologs.out > srna1-homologs.rnaz

In the above command line, 'srna1-homologs.out' is the output file of clustalo and 'srna1-homologs.rnaz' is the output of RNAz. The line ‘Prediction: RNA’ indicates the sRNA was classified as functional sRNA.

```
######################## RNAz   2.1.1 #######################

 Sequences: 3
 Columns: 117
 Reading direction: forward
 Mean pairwise identity:  97.15
 Shannon entropy: 0.03924
 G+C content: 0.50142
 Mean single sequence MFE: -41.96
 Consensus MFE: -42.09
 Energy contribution: -41.43
 Covariance contribution:  -0.66
 Combinations/Pair:   1.09
 Mean z-score:  -3.84
 Structure conservation index:   1.00
 Background model: dinucleotide
 Decision model: sequence based alignment quality
 SVM decision value:   3.41
 SVM RNA-class probability: 0.998597
 Prediction: RNA

######################################################################

>REFSEQ-ID-SRNA1
GAGAGAUGCCCUCCUGGAUGGGGAAUACAGACCUUCGACAAGUCGUAUUAUCCCAGUUC
AGAAGGGCGUUUCUCUUUUUCCCACGCCGCUACCCCUAAAUCACAUCGGUGUAUCCAG
((((((((((((.(((((((((((((((.(((.........)))))))).))))).))))).))))))))))))........((((((.................))))))......   (   -44.83,
z-score =  -4.96, R)
>REFSEQ-ID-HOMOLOG-SRNA1
GAGAGAUGCCCUCCUGGAUGGGGAAUAUAGACCUUCGACAAGUCGUAUUAUCCCAGUUC
AGAAGGGUAUUUCUCUUUUUCCCACGCCGCUACCCCAAAAUCACAUCGGUGGAUCCAG
((((((((((((.(((((((((((((((.(((.........)))))))).))))).))))).))))))))))))).........(((((.................))))).......   (   -40.53,
z-score =  -3.28, R)
>REFSEQ-ID-HOMOLOG-SRNA2
GAGAGAUGCCCUCCUGGAUGGGGAAUAUAGACCUUCGACAAGUCGUAUUAUCCCAGUUC
AGAAGGGUAUUUCUCUUUUUCCCACGCCGCUACCCCAAAAUCACAUCGGUGGAUCCAG
((((((((((((.(((((((((((((((.(((.........)))))))).))))).))))).))))))))))))).........(((((.................))))).......   (   -40.53,
z-score =  -3.28, R)
>consensus
GAGAGAUGCCCUCCUGGAUGGGGAAUAUAGACCUUCGACAAGUCGUAUUAUCCCAGUUC
AGAAGGGUAUUUCUCUUUUUCCCACGCCGCUACCCCAAAAUCACAUCGGUGGAUCCAG
((((((((((((.(((((((((((((((.(((.........)))))))).))))).))))).))))))))))))).........(((((.................))))).......  (-42.09  =
```

-41.43 + -0.66)

5. Run RNAdetect (Chen et al., 2019) with the alignments obtained on step 3 to detect functional sRNAs.

   $ RNAdetect srna1-homologs.out -o srna1-homologs.rnadetect

   In the above command, 'srna1-homologs.out' is the output file of clustalo and 'srna1-homologs.rnadetect is the output of RNAdetect. In the output, the 1 below “ncRNA_decision” indicates that it was classified as functional sRNA.

   Index: window(start,end) Num_Seqs ncRNA_probability ncRNA_decision(P>0.50)
   1: (1,   117) 3 0.977459 1

6. Proceed with the analysis in case this sRNA cantidade is classified as functional sRNAs by at least one of the classifiers (RNAdetect or RNAz).

7. Predict RNA structure with RNAalifold (Bernhart et al., 2008).

   $ RNAalifold --MEA --color --aln --aln-stk -f=C --id-prefix=srna1-structure srna1-homologs.out

   In the command line, ‘id-prefix’ is the prefix for creating the output files and ‘srna1-homologs.out’ is the alignment file. Supplementary Figure 7 presents the sRNA structure created by RNAalifold.

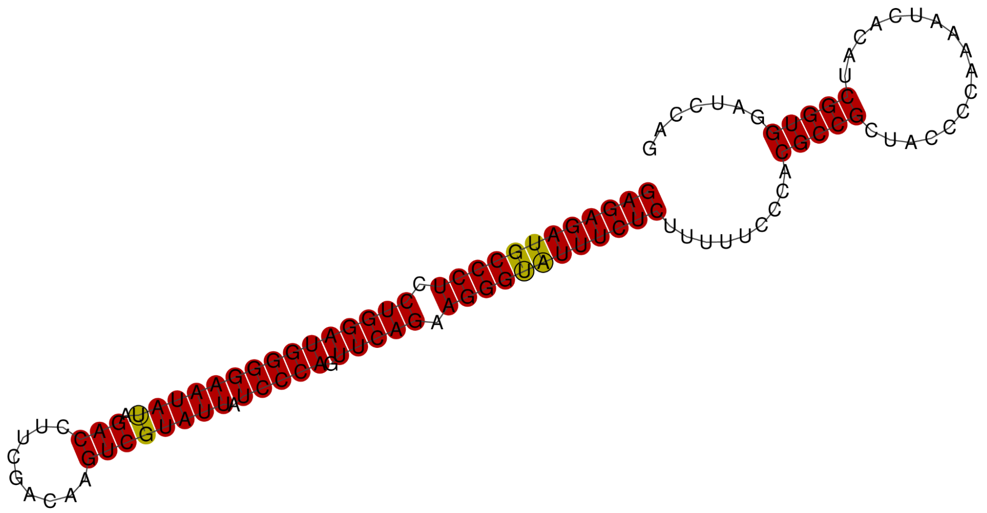

Supplementary Figure 7 - sRNA structure

8. Predict sRNA targets with CopraRNA.

```
$ CopraRNA2.pl -srnaseq  srna1-homologs.fa -ntup 200 -ntdown 100 -region 5utr
-topcount 200 -cores 8 --websrv
```

In the above command, 'srna1-homologs.fa' is the multi fasta file with the three homolog sRNA. Select the fifteen best-ranked interactions from “coprarna_websrv_table.csv” predicted with a p-value <0.01, as suggested in (Wright and Georg, 2018).

Rank	CopraRNA p-value	CopraRNA fdr	Locus Tag	Energy kcal/mol	IntaRNA p-value	Position mRNA	Position ncRNA
1	0	0	ce_rs00795	-158.49	0	82 -- 198	1 -- 117
2	6.938E-05	0.0578	ce_rs10235	-24	0.006048	19 -- 69	76 -- 116
3	0.001096	0.5368	ce_rs03990	-14.9	0.052554	217 -- 270	76 -- 116
4	0.001289	0.5368	ce_rs07695	-20.56	0.013131	12 -- 54	76 -- 117
5	0.001657	0.552	ce_rs02350	-23.63	0.006563	202 -- 233	81 -- 116
6	0.002118	0.5881	ce_rs09315	-16.38	0.036069	271 -- 300	80 -- 111
7	0.002493	0.5883	ce_rs12615	-13.77	0.070397	57 -- 79	81 -- 117
8	0.003151	0.5883	ce_rs13325	-15.69	0.042903	102 -- 129	85 -- 116

9	0.003178	0.5883	ce_rs01160	-14.67	0.055748	268 -- 288	76 -- 96
10	0.005524	0.8658	ce_rs03900	-16.42	0.035674	200 -- 226	78 -- 106
11	0.007284	0.8658	ce_rs10785	-21.28	0.011118	71 -- 109	78 -- 117
12	0.00855	0.8658	ce_rs10090	-8.29	0.300378	61 -- 86	84 -- 116
13	0.008677	0.8658	ce_rs07750	-15.06	0.050448	130 -- 160	80 -- 117
14	0.008799	0.8658	ce_rs04940	-17.1	0.0301	113 -- 139	84 -- 108
15	0.009027	0.8658	ce_rs07770	-13.42	0.077212	81 -- 101	78 -- 95

9. Calculate adjusted p-values using the Beijamini-Hochberg correction from the R package stats, method p.adjust (p.adjust). 'Pvalue_predicted' is the set of p-values from CopraRNA and 'correct_pvalue' is the set of corrected p-values.

   $ corrected_pvalue <- p.adjust(pvalue_predicted, method = "BH", n = length(pvalue_predicted))

Rank	CopraRNA p-value	Adjusted p-value	CopraRNA fdr	Locus Tag	Energy kcal/mol	IntaRNA p-value	Position mRNA	Position ncRNA
1	0	0.00E+00	0	ce_rs00795	-158.49	0	82 -- 198	1 -- 117
2	6.938E-05	1.94E-03	0.0578	ce_rs10235	-24	0.006048	19 -- 69	76 -- 116
3	0.001096	7.04E-03	0.5368	ce_rs03990	-14.9	0.052554	217 -- 270	76 -- 116
4	0.001289	7.30E-03	0.5368	ce_rs07695	-20.56	0.013131	12 -- 54	76 -- 117
5	0.001657	7.84E-03	0.552	ce_rs02350	-23.63	0.006563	202 -- 233	81 -- 116
6	0.002118	8.42E-03	0.5881	ce_rs09315	-16.38	0.036069	271 -- 300	80 -- 111
7	0.002493	8.86E-03	0.5883	ce_rs12615	-13.77	0.070397	57 -- 79	81 -- 117
8	0.003151	9.67E-03	0.5883	ce_rs13325	-15.69	0.042903	102 -- 129	85 -- 116
9	0.003178	9.68E-03	0.5883	ce_rs01160	-14.67	0.055748	268 -- 288	76 -- 96
10	0.005524	1.25E-02	0.8658	ce_rs03900	-16.42	0.035674	200 -- 226	78 -- 106
11	0.007284	1.47E-02	0.8658	ce_rs10785	-21.28	0.011118	71 -- 109	78 -- 117
12	0.00855	1.64E-02	0.8658	ce_rs10090	-8.29	0.300378	61 -- 86	84 -- 116

13	0.008677	1.65E-02	0.8658	ce_rs07750	-15.06	0.050448	130 -- 160	80 -- 117
14	0.008799	1.67E-02	0.8658	ce_rs04940	-17.1	0.0301	113 -- 139	84 -- 108
15	0.009027	1.70E-02	0.8658	ce_rs07770	-13.42	0.077212	81 -- 101	78 -- 95

References

Bernhart, S. H., Hofacker, I. L., Will, S., Gruber, A. R., and Stadler, P. F. (2008). RNAalifold: improved consensus structure prediction for RNA alignments. *BMC Bioinformatics* 9, 474. doi:10.1186/1471-2105-9-474.

Chen, C.-C., Qian, X., and Yoon, B.-J. (2019). RNAdetect: efficient computational detection of novel non-coding RNAs. *Bioinformatics* 35, 1133–1141. doi:10.1093/bioinformatics/bty765.

Gruber, A. R., Findeiß, S., Washietl, S., Hofacker, I. L., and Stadler, P. F. (2010). RNAz 2.0: improved noncoding RNA detection. *Pac. Symp. Biocomput.*, 69–79. Available at: https://www.ncbi.nlm.nih.gov/pubmed/19908359.

Kalvari, I., Nawrocki, E. P., Argasinska, J., Quinones-Olvera, N., Finn, R. D., Bateman, A., et al. (2018). Non-Coding RNA Analysis Using the Rfam Database. *Curr. Protoc. Bioinformatics* 62, e51. doi:10.1002/cpbi.51.

Lott, S. C., Schäfer, R. A., Mann, M., Backofen, R., Hess, W. R., Voß, B., et al. (2018). GLASSgo - Automated and Reliable Detection of sRNA Homologs From a Single Input Sequence. *Front. Genet.* 9, 124. doi:10.3389/fgene.2018.00124.

Nawrocki, E. P., and Eddy, S. R. (2013). Infernal 1.1: 100-fold faster RNA homology searches. *Bioinformatics* 29, 2933–2935. doi:10.1093/bioinformatics/btt509.

p.adjust Available at: https://www.rdocumentation.org/packages/stats/versions/3.6.2/topics/p.adjust [Accessed August 16, 2020].

Sievers, F., Wilm, A., Dineen, D., Gibson, T. J., Karplus, K., Li, W., et al. (2011). Fast, scalable generation of high-quality protein multiple sequence alignments using Clustal Omega. *Mol. Syst. Biol.* 7. Available at: https://www.embopress.org/doi/abs/10.1038/msb.2011.75.

Wright, P. R., and Georg, J. (2018). Workflow for a Computational Analysis of an sRNA Candidate in Bacteria. *Methods Mol. Biol.* 1737, 3–30. doi:10.1007/978-1-4939-7634-8_1.

Wright, P. R., Richter, A. S., Papenfort, K., Mann, M., Vogel, J., Hess, W. R., et al. (2013). Comparative genomics boosts target prediction for bacterial small RNAs. *Proc. Natl. Acad. Sci. U. S. A.* 110, E3487–96. doi:10.1073/pnas.1303248110.
